# Analysis of per- and polyfluoroalkyl substances (PFAS) removal with activated carbon using ^19^F nuclear magnetic resonance spectroscopy

**DOI:** 10.1039/d5ra06437f

**Published:** 2026-01-26

**Authors:** G. F. Earl, L. A. Stevens, W. Li, C. E. Snape

**Affiliations:** a Department of Chemical and Environmental Engineering, Faculty of Engineering, University of Nottingham Nottingham NG7 2RD UK

## Abstract

Per- and polyfluoroalkyl substances (PFAS) are a toxic and environmentally persistent class of chemicals that are associated with a myriad of adverse health effects in humans. Activated carbon is an effective material for the removal of PFAS at water treatment plants, but there is a necessity to find new, superior coal-derived and biomass-derived forms, due to the increasing accumulation of PFAS in treatable water sources and the need to shift to biomass-derived adsorbent usage to offset the carbon emissions released during their production. Additionally, current methods of PFAS detection and quantification utilise expensive and laborious analytical techniques, such as liquid-chromatography with tandem mass spectrometry. In this study, four commercial activated carbons (CAC-1, CAC-2, CAC-3 and CAC-4) had their chemical/textural properties characterised and had their PFAS removal performances investigated using four different PFAS types (PFBS, PFHxA, PFOA AND PFOS), with detection and quantification conducted with ^19^F nuclear magnetic resonance (^19^F-NMR). The adsorption results revealed the overall ranking of adsorbents by performance as CAC-4 > CAC-3 > CAC-2 > CAC-1, with CAC-4 being the highest ranking adsorbent for removing all four PFAS, due to a high surface area coupled with superior mesoporosity as well as containing a higher concentration of surface oxygen-containing functional groups, which fortify adsorption with favourable hydrophobic interactions and electrostatic interactions, respectively. Moreover, the effective use of ^19^F-NMR for the detection and quantification of total PFAS content after activated carbon treatment was demonstrated, with the use of inexpensive internal standards and a near-complete lack of sample clean-up. As such, quantitative ^19^F-NMR can be a viable, low-cost alternative to current PFAS analytical techniques for the screening of new adsorbent media.

## Abstract 2

^19^F NMR is used to screen and rank activated carbons for PFAS removal, allowing direct quantification of PFOS, PFOA, PFHxA and PFBS to identify the most effective adsorbent.

## Graphical abstract

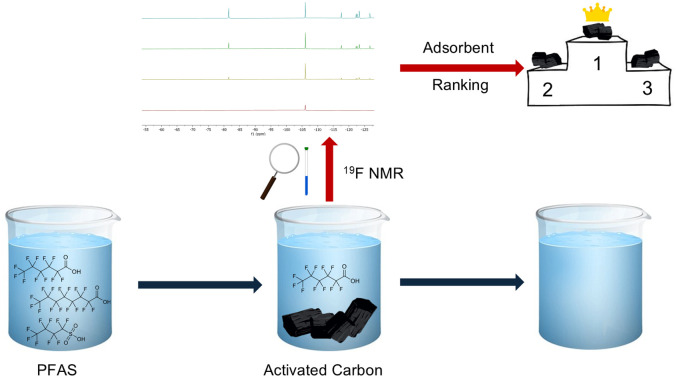

## 1 Introduction

Per- and polyfluoroalkyl substances (PFAS) are a family of synthetic fluorinated aliphatic substances, typically characterised by the presence of a terminal perfluorinated methyl group (–CF_3_), a perfluorinated carbon chain of variable length (–CF_2_–)_*n*_ and a terminal polar functional group.^1^ These structural features enable PFAS molecules to exhibit high stability, as well as hydrophobic and lipophobic properties.^2^ These unique and useful properties have led to their application in a wide range of consumer goods, such as fire-fighting foams, cleaning products, water-resistant fabrics, non-stick cookware and food packaging.^3^ The C–F bond is the strongest covalent bond in organic chemistry (488 kJ mol^−1^), thus enabling PFAS to be highly resistant to degradation.^4^ Additionally, the low polarisability of the fluorine atoms engenders the lipophobic/hydrophobic properties, whilst the hydrophilic polar head groups give rise to water-solubility, enabling efficient mobility in aqueous environments. The combination of these properties has contributed to the environmental persistence of PFAS and their tendency to bioaccumulate in living organisms.^5^ The OECD have recorded and categorised over 4700 PFAS analogues synthesised since the 1950s.^6^ Moreover, the United States Environmental Protection Agency has identified more than 9000 PFAS, which are compiled in their CompTox database, mostly consisting of fluorinated surfactants and polymers.^7^

PFAS can be grouped into different subclasses according to their chain-length and their terminal functional group.^8^ Perfluoroalkyl carboxylic acids (PFCA) are classed as “long-chain” PFAS when their carbon chain contains greater than seven carbon atoms. In contrast, perfluoroalkyl sulfonic acids (PFSA) are referred to as “long-chain” when their carbon chain contains greater than five carbon atoms. Similarly, “short-chain” PFAS envelope PFCAs with a carbon chain containing fewer than eight carbon atoms and PFSAs containing fewer than six carbon atoms. The PFAS that are the focus of this study that span these subclasses are the long-chain perfluorooctanoic acid (PFOA), perfluorooctane sulfonic acid (PFOS), and the short-chain perfluorohexanoic acid (PFHxA) and perfluorobutane sulfonic acid (PFBS) (Table 1). Human exposure to PFAS has been linked to a variety of health issues, including decreased fertility, high blood pressure in pregnant women, low birth-weights, accelerated puberty, increased risk of prostate/kidney/testicular cancers, weakening of the immune system, increased cholesterol levels and interference with the body's natural hormones.^2^ Consequently, UK water companies are required to regularly test for 47 different types of PFAS, with each individual PFAS being restricted from exceeding 100 ng L^−1^ in potable water. This threshold is significantly less rigorous than the restrictions set in place by the USA, where a limit of 4 ng L^−1^ has been imposed for the two most prevalent PFAS (PFOA and PFOS). Similarly, the Drinking Water Directive (EU) has stated that 20 of the most commonly used PFAS must not collectively surpass a limit of 100 ng L^−1^. In England, PFAS has been detected in samples from drinking water sources at 17 of 18 water companies, with 3.8% of the samples testing positive. The maximum concentration of one particular type of PFAS (PFOS) was detected at 1.86 µg L^−1^ in untreated water, which is more than 18 times greater than the limit for potable water. PFOA and PFOS were the two most widely used PFAS in the UK, and it took 40 years to gather sufficient data to regulate their production and use. This led to less-effective short-chain PFAS being used as substitutes in industrial processes/manufacturing, where greater quantities are required to achieve the same results, and they are significantly more difficult to remove during water treatment. Although short-chain PFAS are thought to bioaccumulate less than long-chain PFAS, they will continue to persist in the environment, and their health implications have not been studied to the same extent as long-chain PFAS.

**Table 1:** Names, structures and chemical properties of the four PFAS types used in this study

Compound name	Molecular structure	Acronym	MW (g mol^−1^)	log *K*_ow_	p*K*_a_
Perfluorobutane sulfonic acid	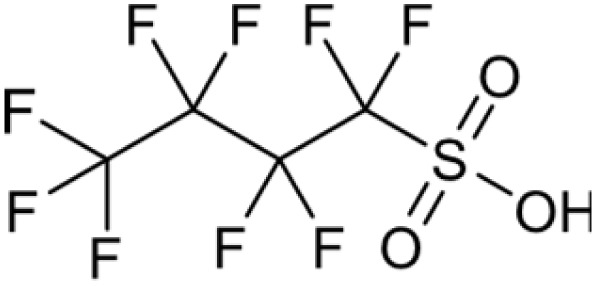	PFBS	300.09	2.63	−3.31
Perfluorooctane sulfonic acid	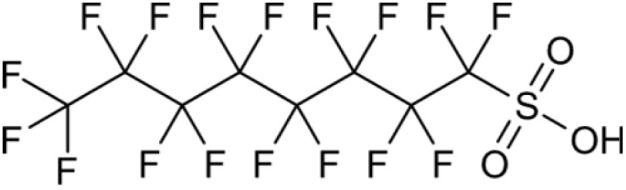	PFOS	500.13	5.43	−3.32
Perfluorohexanoic acid	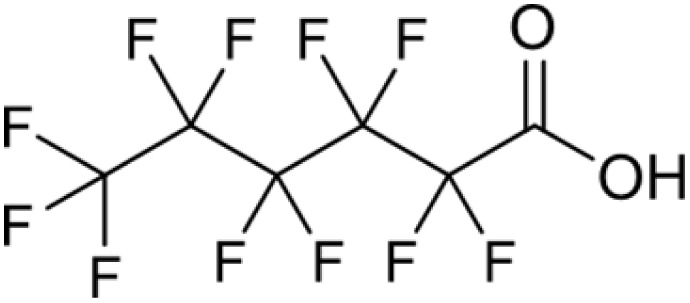	PFHxA	314.05	3.71	−0.78
Perfluorooctanoic acid	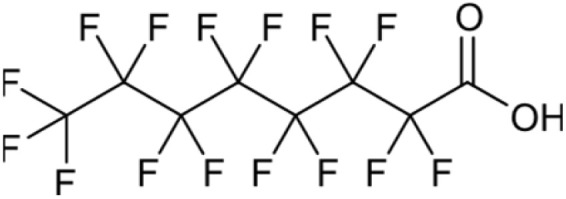	PFOA	414.07	5.11	−4.20

At present, there are a variety of treatment techniques capable of removing PFAS, including adsorption,^9–11^ electrochemical oxidation,^12–14^ reverse osmosis,^15,16^ photocatalysis,^17–19^ biological remediation,^20^ nanofiltration^21^ and foam fractionation.^22^ Of these treatment technologies, adsorption is the most widely used and established method of PFAS removal, either as a point-of-use application or as an integrated tertiary treatment step in a water treatment plant. Activated carbon (AC) is the most popular removal technique, ascribed to low production costs, sound performance and simplicity in its application. AC used for PFAS removal is sold in a few different forms: powdered activated carbon (PAC), granular activated carbon (GAC), pelletised activated carbon (pelletised AC) and activated carbon fibres (ACF).^23^ There have been reports of excellent removal capacities of long-chain PFAS for all the above-mentioned types of AC due to their favourable physicochemical properties, namely the attractive hydrophobic interaction between the AC graphitic surface and the fluorinated tail of the long-chain PFAS. For short-chain PFAS removal, AC removal performance is decreased due to the increased hydrophilicity of the PFAS as the carbon chain decreases in length.^24^ Of the different forms of AC, GAC is the most commercially viable as it is capable of being regenerated through either physical or chemical reactivation, thus enabling the recycling of the same GAC many times over, although the industrial techniques for reactivating PFAS-laden GAC are in their infancy.^25,26^

Detection and quantification of PFAS is typically carried out using liquid-chromatography with mass spectrometry (LC-MS) techniques, but only a fraction of all PFAS can be detected this way.^27^ The insubstantial identification of PFAS is due to a variety of reasons, including poor analyte recoveries during sample extraction, substandard ionisation efficiencies during LC-MS, a paucity of isotopically pure internal standards, and some PFAS not presenting characteristic MS fragmentation patterns, which are key to their identification.^28,29^ Additionally, the high purchase price, maintenance/operational costs, lack of automation, limited sample throughput, infrastructure requirements and matrix effects make LC-MS an analytical technique with complications.

There is a pressing need to develop more rapid and lower cost methods of PFAS detection and quantification than LC-MS/MS that is capable of detecting all types of PFAS individually or the total PFAS in a sample. As environmental bodies continue to lower regulatory limits for PFAS and demand for testing increases with more PFAS compounds being targeted, alternative or complementary technologies will need to be researched to meet the screening requirements. This will allow for a more varied representation of the performance of ACs in PFAS removal, as well as aiding in the assessment of new remediation technologies targeting lesser-known PFAS. ^19^F nuclear magnetic resonance (^19^F-NMR) spectroscopy has been reported as a valuable technique for total PFAS detection and quantification.^30–32^ The terminal perfluorinated methyl group (–CF_3_) upon the alkyl chain of the PFAS produces a signal (−82 ppm) which is the same for each type of PFAS. This signal can quantify the total PFAS in a mixed sample or the concentration of a PFAS in a single PFAS sample.

Although ^19^F-NMR cannot reach the limits of detection (LOD) achieved by LC-MS (LC-MS/MS can have LOD as low as ppt), the technique does present some significant advantages, including rapid sample analysis, affordable operational costs, facile sample preparation with a lack of matrix effects and the method is capable of quantification using inexpensive reference standards. Addition of a fluorinated internal standard facilitates quantification, for example, 4,4′-difluorobenzophenone (DFBP) may be added to the NMR sample and the intensity of the signal provided by the 2 fluorine nuclei of the molecule can be used as a basis to calculate the total PFAS concentration by comparison with the intensity of the signal provided by the 3 fluorine nuclei of the terminal –CF_3_ peak. Camdzic *et al.* demonstrated total PFAS quantification of wastewater samples using ^19^F-NMR and reported their method detected higher total PFAS quantities than both LC-MS and total oxidisable precursor (TOP) assay analyses, which suggested the underreporting of PFAS from the two more researched quantitative analytical methods.^32^ Moreover, Gauthier *et al.* measured the ^19^F-NMR spectra of hundreds of different fluorinated reference compounds in order to build a database to enable quantitative ^19^F-NMR analyses of environmental samples containing fluorinated compounds that would be missed by routine mass spectrometry methods, closing the gap in total fluorine mass balance in environmental samples.^33^

The aim of this study was to demonstrate that ^19^F-NMR can be used as a practical tool for the analysis of aqueous PFAS and, therefore, the rapid selection of new variants of PFAS-removing technologies, like activated carbon. The ^19^F-NMR spectra for a series of PFAS with contrasting chain lengths and head groups and extensive characterisation data for commercial activated carbons, are presented. The study demonstrates that initial testing of novel adsorbents for PFAS removal may be carried out with ^19^F-NMR, significantly lowering expenditure and reducing experimental time. Additionally, the novel method of PFAS quantification was utilised to compare the adsorption performances of the four commercial ACs in removing PFAS.

## 2 Materials and methods

### 2.1. Materials

The four selected ACs (Table 2) are all commercially available granular activated carbons. All the ACs were prepared *via* pyrolysis of the precursor feedstock, followed by physical steam activation of the resulting char.

**Table 2:** Names, forms and precursor materials of the four adsorbents

Form	Name	Precursor
Granular	CAC-1	Bituminous coal
Granular	CAC-2	Coconut shell
Granular	CAC-3	Agglomerated bituminous coal
Granular	CAC-4	Bituminous coal

Two PFCAs and two PFSAs were selected as the adsorbates for the batch adsorption experiments: perfluorohexanoic acid (PFHxA), perfluorooctanoic acid (PFOA), perfluorobutane sulfonic acid (PFBS) and perfluorooctane sulfonic acid (PFOS). All PFAS were purchased from Sigma-Aldrich, United Kingdom. The details of the four PFAS are presented in Table 1. Stock solutions of each PFAS were prepared using ultrapure distilled water from an Arium® Pro Ultrapure Lab Water System. Each PFAS solution was made as a master batch at 241.5 µM. Deuterated methanol (99%), chromium(iii) acetylacetonate (99.99%) and 4,4′-difluorobenzophenone were acquired from Sigma-Aldrich, United Kingdom.

### 2.2. Textural analysis and pH_PZC_

The specific surface area and pore size distribution of the milled ACs were evaluated by both CO_2_ and N_2_ adsorption–desorption isotherms at −196.15 °C and 0 °C, respectively, using an ASAP™ 2420 Accelerated Surface Area and Porosimetry System, Micromeritics. Brunauer–Emmett–Teller (BET) theory was used to analyse the N_2_ isotherm at −196.15 °C to calculate the specific surface area, utilising a relative pressure range of 0.08 to 0.15, whilst the pore volumes and pore size distribution were determined using density functional theory (NLDFT) for carbon slit pores by combined analysis of the N_2_ and CO_2_ adsorption isotherms (MicroActive Software V5.0, Micromeritics).

Bright-field transmission electron microscopy (TEM) was performed at 200 kV on a JEOL 2100F TEM with a Gatan Model 1095 K3-IS direct electron detection camera (point resolution 0.23 nm). Samples were deposited on holey carbon on copper 200 mesh TEM grids (Agar Scientific) by bringing the dry sample into contact with the grid. TEM is a relevant tool to reveal the profile of the polyaromatic layers which form the framework of the ACs.

Scanning electron microscopy (SEM) was performed with a Zeiss Crossbeam 550 FIB-SEM. Dry samples of the AC were attached to an SEM stub using conductive carbon cement. Samples were fractured and cut with a scalpel to view the internal structure and subsequently coated with ∼11 nm of iridium at 10 mA for 60 s. Images were acquired at set magnifications (500–2500×), and the imaging conditions remained constant. The magnification of SEM is not powerful enough to reveal meso- or micropores, but it will show the macroporosity, which does play a part in PFAS adsorption.

The point of zero charge (pH_PZC_) of each AC was determined *via* the pH drift method. The pH_PZC_ indicates the pH at which the net surface charge of the AC is equal to zero. The net surface charge of the AC can influence the electrostatic interactions between the adsorptive and the adsorbate. A total of 9 solutions of NaCl (0.01 M, 50 mL) were prepared with the pH ranging from 2–10. pH modification was achieved by adding HCl (0.1 M) or NaOH (0.1 M). To each solution, pulverised AC was added (0.2 g) and the mixtures were agitated on an orbital shaker for 24 hours. The final pH was recorded with the FiveEasy Plus pH Meter FP20. A plot of the initial pH (pH_initial_) *versus* the final pH (pH_final_) was recorded and the point at pH_initial_ is equal to pH_final_ was deemed to be the pH_PZC_. Hydrochloric acid, sodium chloride and sodium hydroxide were acquired from Sigma-Aldrich, United Kingdom.

The X-ray photoemission spectra of the AC were obtained using a Thermo Fisher Scientific K-Alpha X-ray photoelectron spectrometer (XPS) to study the extent of the presence of surface oxygen-containing functional groups that may partake in adsorptive processes. An achromatic X-ray radiation of 1486.6 eV (AlKα) was used for each AC. All spectra were recorded using a pass energy of 20 eV, and the spot size is approximately 400 µm. To determine the various oxygen-containing functional groups, high resolution scans of the C 1s have been collected for each AC using 0.1 eV step sizes. The XPS spectra were deconvoluted using the CasaXPS software. Baseline corrections were made using a Doniach–Sunjic–Shirley (DSS) line shape.^34,35^

Thermogravimetric analysis (TGA) was performed using a TA-Instruments Q500 Thermogravimetric Analyser. A sample of ∼20 mg was packed into a platinum crucible, which had been counterbalanced by the same crucible before sample loading. The proximate analysis was conducted under a nitrogen and air flow (100 mL min^−1^, each) from 25 °C to 900 °C, first heating under a nitrogen atmosphere to 915 °C, cooling to 815 °C and switching to air flow for combustion of the fixed carbon. Each AC was analysed 5 times.

Ultimate analysis was performed on the ACs using a LECO CHNS 628 Elemental Analyser to determine carbon, nitrogen, hydrogen, sulphur and oxygen (by difference) compositional data.

### 2.3. Batch adsorption tests

The AC samples were milled and sieved to a size of ≤150 µm. The dosage for each AC in batch adsorption isotherm experiments were set at fixed masses of 40, mg L^−1^, 80 mg L^−1^, 160 mg L^−1^, 320 mg L^−1^ and 640 mg L^−1^. Initial tests were performed with just PFOA at 100 mg L^−1^ (241.5 µM), therefore subsequent solutions of PFBS, PFHxA and PFOS were prepared to 241.5 µM in individual solutions in ultrapure water. All batch adsorption experiments were conducted with an individual PFAS type (no mixtures). Blank PFAS solutions and PFAS solutions dosed with the AC were prepared in high-density polyethylene (HDPE) bottles (250 mL) and were continuously agitated at 150 rpm on a Fisherbrand™ Digital Orbital Shaker at 25 °C. The ultrapure water used for the PFAS solutions had a stable pH measured at pH 6 (±0.2). Blank tests went under identical procedures to the active tests, but without the addition of any AC. After 48 hours, 2 mL aliquots were taken and filtered with a 33 mm diameter 0.45 µm Fisherbrand™ Syringe Filter and the 300 µL filtrate was combined with 300 µL of the standard spiked (4,4′-difluorobenzophenone) deuterated methanol in an NMR tube. Additionally, Cr(acac)_3_ was added to the deuterated methanol and made up to 8 mg mL^−1^. This was done because half the NMR samples were made up with deuterated methanol (0.3 mL) and a Cr(acac)_3_ concentration of 4 mg mL^−1^ was reported to be optimal to reduce spin-lattice relaxation during the NMR experiments, whilst mitigating excessive line broadening.^36^ The concentration of the PFAS and the internal standard in the blank sample were equimolar (120.8 µM) in each NMR sample. A random sample from each experiment had 3 replicates taken. Batch sorption equilibration tests were also performed, with samples taken after 1, 4, 8 and 14 days, with 14 days having been shown to be suitable for equilibration in another study.^37^ Batch sorption test data were fitted with the non-linear Langmuir, Freundlich and Sips equations.^38^ The non-linear Langmuir equation can be expressed as:1
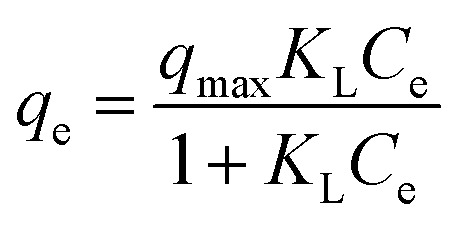
where *q*_e_ (mg g^−1^) is the equilibrium concentration of the PFAS adsorbed on the solid phase, *C*_e_ (mg L^−1^) is the equilibrium concentration of the PFAS in the liquid phase, *K*_F_ is the Freundlich distribution coefficient in (mg g^−1^)/(mg g^−1^)^−1/*n*^ and 1/*n* is the Freundlich exponent which indicates the intensity of adsorption. The non-linear Freundlich equation can be expressed as:2
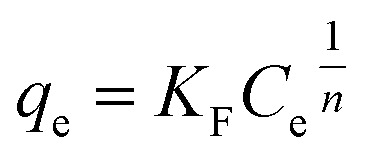
where *q*_max_ is the maximum adsorption capacity (mg g^−1^) and *K*_L_ is the Langmuir constant related to the affinity of the binding sites (L mg^−1^).

The non-linear Sips equation can be expressed as:3
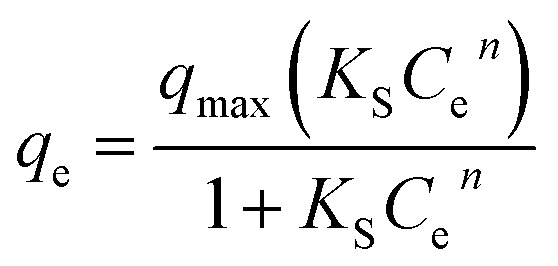
where *K*_S_ is the Sips equilibrium constant (L mg^−1^) and *n* is the dimensionless heterogeneity factor.

The PFAS removal percentage (% *R*) was also calculated for each AC according to eqn (4):4
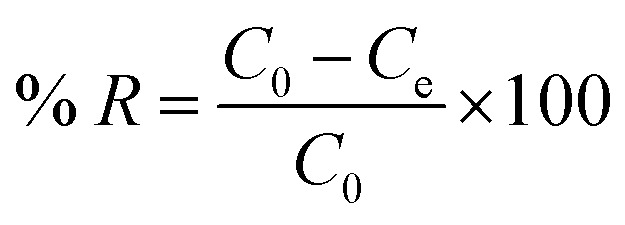


Adsorption measurements were performed in HDPE bottles (250 mL), with all four ACs dosed at 640 mg L^−1^ and all PFAS dosed at 241.5 µM. All solutions were agitated at 150 rpm at 20 °C. Samples were taken at 15, 30, 60, 90, 120 and 180 min from each solution.

The extent of PFAS adsorption was calculated with the following equation:5
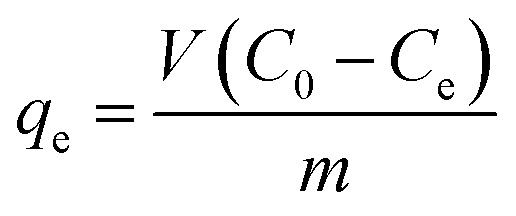
where *V* (L) is the volume of solution, *m* is the mass of AC (g), *C*_0_ is the initial PFAS concentration and *C*_e_ is the equilibrium concentration of PFAS corresponding to *q*_e_.

Adsorption kinetics studies were conducted for PFBS, PFHxA, PFOA and PFOS on CAC-1, CAC-2, CAC-3 and CAC-4 using the pseudo-first-order and pseudo-second-order kinetic models. The pseudo-first-order model can be expressed as:

6 ln(*q*_e_ − *q*_*t*_) = ln(*q*_e_) − *K*_1_*t*

The pseudo-second-order model can be expressed as:7
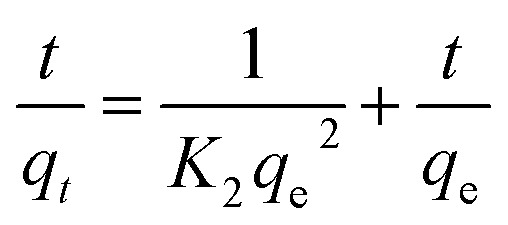
where *q*_*t*_ (mg g^−1^) is the quantity of PFAS adsorbed on the AC at time *t* and *q*_e_ (mg g^−1^) is the quantity of PFAS adsorbed at equilibrium. *K*_1_ (min^−1^) and *K*_2_ (mg [g min]^−1^) correspond to the rate constants for the pseudo-first-order and pseudo-second-order rate equations, respectively.^39^

### 2.4. PFAS detection and quantification

^19^F-NMR spectra were collected on a Bruker 500 MHz spectrometer equipped with a broad-band tuneable liquid-nitrogen cooled cryoprobe and room temperature TXI probe. The ^19^F-NMR analysis was performed at 298 K, the spectral width was −145 to −30 ppm, the acquisition time was 2.8 s, the relaxation delay was 2 s, a 90° pulse width of 14 µs and a total of 1024 transients for each sample, resulting in about 1.5 h of experimental time. The ^19^F-NMR spectra were processed using MestreNova™ software. A random sample from each experiment had its ^19^F-NMR spectra acquired in triplicate.

The correct solvent choice was vital for the development of the ^19^F-NMR method. Since the deuterated solvent was to be mixed in a 1 : 1 ratio, on a volumetric basis, with the treated PFAS solutions for the NMR experiments, the chemical conditions of the final NMR sample solution had to be tailored correctly. Deuterated chloroform (CDCl_3_) was not viable since two distinct layers formed upon mixing the solvent and the PFAS solution, possibly due to the higher concentrations of PFAS used in this study (potential hemimicelle/micelle formation). After trials were conducted with deuterated water (D_2_O) and the PFAS solution in a 1 : 1 ratio, with the mixture being miscible, however, the addition of Cr(acac)_3_ as a paramagnetic relaxation agent was not possible due to its insolubility in H_2_O/D_2_O. Lastly, CD_3_OD was trialled, which was miscible with the PFAS solution. The Cr(acac)_3_ was soluble at the optimal dose rate, which was 4 mg mL^−1^. In the subsequent NMR experiments, signal locking was achieved, and the spectra displayed well-resolved signals that were suitable for quantification based on their integrals.

S : N experiments were conducted with PFOA in deuterated methanol (CD_3_OD) with Cr(acac)_3_ with no added standard to ensure the terminal –CF_3_ peak could be analysed and compared with the spectral noise with no influence from other peaks. The relaxation delay, *D*_1_, was set to 2 s to ensure total relaxation of all fluorine nuclei. The S : N in future quantitative NMR experiments would need to be greater than or equal to 3 : 1 to ensure result validity, and so the lowest concentration of PFOA that corresponded to this ratio was found to be 1 mg L^−1^ at 1024 scans (Fig. S8). In all subsequent adsorption isotherm and kinetics experiments, the S : N noise ratio was always >10.

## 3 Results and discussion

### 3.1. Activated carbon characterisation

Thorough textural analysis of AC plays a vital role in understanding the adsorptive mechanisms that occur during pollutant removal. ACs can be derived from various sources, with the final product exhibiting different chemical and structural properties, depending on the source material and the conditions used in the production. The moisture, volatile matter, fixed carbon and ash weight percentages are presented in Table 3. Ash content can decrease the effectiveness of adsorption, thus, proximate analysis is a relevant tool for understanding why some ACs are not performing as expected, for example, Reid *et al.* highlighted that removing acid-soluble impurities, including ash, led to a 100 m^2^ g^−1^ gain in BET surface area and improved accessibility to micropores which led to an enhancement in PFAS removal performance of the ACs.^40^

**Table 3:** Proximate analysis data for the four adsorbents (dry basis)

Sample	Proximate analysis (as received, wt%)		
	Volatile matter	Fixed carbon	Ash
CAC-1	3.52	81.10	15.32
CAC-2	5.49	86.26	8.14
CAC-3	3.87	89.27	6.90
CAC-4	2.32	85.83	11.92

It is important to quantify surface area and pore structure to interpret adsorption performance. All ACs exhibited relatively high BET surface areas, and higher values generally aligned with greater PFAS uptake (Table 4). Although CAC-3 had the largest specific surface area (1165 m^2^ g^−1^), CAC-4 possessed the greatest total pore volume and a larger proportion of mesopores, which likely enhanced PFAS accessibility within the carbon matrix. Bituminous-coal carbons commonly contain both micro- and mesopores, and CAC-4 followed this expected pattern.

**Table 4:** Textural properties and chemical properties of the ACs. The O content was calculated by difference from the total amount of other elements on a dry, ash-free basis

Sample	Textural properties						pH_PZC_	Chemical compositions [wt%]				
	*S*_BET_ (m^2^ g^−1^)	*V*_total_ (cm^3^ g^−1^)	*V*_ultramicro_ (cm^3^ g^−1^)	*V*_micro_ (cm^3^ g^−1^)	*V*_meso_ (cm^3^ g^−1^)	Average pore diameter (nm)		C	N	H	S	O
CAC-1	1021	0.471	0.195	0.361	0.103	1.85	6.9	73.2	0.8	0.6	0.9	24.5
CAC-2	898	0.411	0.162	0.322	0.085	1.83	8.9	80.6	0.8	1.3	0.6	16.7
CAC-3	1051	0.463	0.189	0.384	0.076	1.76	7.6	76.3	0.5	2.0	0.8	20.4
CAC-4	975	0.538	0.166	0.339	0.193	2.21	7.8	79.9	0.5	0.9	0.8	17.9

In contrast, CAC-1 and CAC-3 were strongly microporous, with >75% of pore volume attributed to micropores. CAC-2, derived from coconut shell, also showed a predominantly microporous structure (∼75%), consistent with the narrow pore size distribution typically reported for coconut-shell activated carbons. The relative concentrations of carbon and oxygen-containing functional groups as well as the oxygen to carbon ratio were studied with XPS *via* the deconvolution of the C 1s spectra, presented in Table 5 and Fig. S5. Of the four ACs, CAC-4 exhibited the greatest O : C ratio, as well as exhibiting the greatest concentration of C–O and COO functional groups. An attempt was made to semi-quantify the nitrogen content on the ACs *via* the N 1s XPS spectra. Still, the surface nitrogen content on all four ACs was too low to enable the deconvolution of the spectra. Electrostatic interaction between the PFAS and the surface functional groups of the AC is one of the main adsorptive processes that occurs. Carbon adsorbents exhibiting a greater concentration of positively charged nitrogen-containing functional groups, such as pyridinic-N and graphitic-N, will aid PFAS adsorption through facilitating π–π stacking interactions.^41^

**Table 5:** Summary of assigned XPS deconvolution peaks/areas and apparent (O : C) ratio obtained from C 1s deconvolution

Deconvolution peaks	Peak label	CAC-1	CAC-2	CAC-3	CAC-4
C–C low (%)	I	6.8	6.5	6.9	6.4
C–C primary (%)	II	83.6	83.8	84.1	79.2
C–C high (%)	III	1.7	2.1	1.7	4.2
C–O (%)	IV	5.0	4.5	4.3	6.9
C=O (%)	V	0.8	0.8	0.9	0.4
COO (%)	VI	2.1	2.2	2.0	2.9
Pi–pi*	VII	0	0	0	0
O : C (C 1s)		0.10	0.10	0.09	0.13

The ACs were examined by both SEM and TEM. SEM micrographs of the four ACs (Fig. S6) clearly shows the pitted and cracked surface morphology as well as the presence of grains, indicating the pore development that occurred during the pyrolysis and activation stages of the AC preparation, thus engendering a ruptured surface. TEM images did not reveal any micro- or mesopores, but did depict a layered structure, particularly in CAC-3 (Fig. S7).

### 3.2. ^19^F-NMR method development

It is possible to accurately quantify PFAS concentrations using ^19^F NMR, but there are drawbacks as well as advantages associated with this approach. The method was able to follow the progress of adsorption, with the –CF_3_ signal intensity gradually decreasing relative to the internal standard signal over time. In previous studies, the accuracy of concentration measurements has been assessed using the signal-to-noise ratio, which must exceed 3 for the limit of detection (LOD) and 10 for the limit of quantification (LOQ), the former being selected as the absolute minimum threshold because a peak three times larger than the standard deviation of the noise is unlikely to arise from random fluctuations, thereby minimising false positives.^36^ In the present study, the determination of the signal-to-noise ratio was based on the –CF_3_ peak at approximately −82 ppm, as this signal was selected for integration and direct comparison with the integral of the internal standard peak in subsequent experiments.

The resulting LOD of approximately 1 mg L^−1^ indicates that, in its current form, the method is not suitable for direct analysis of environmental water samples, where PFAS concentrations are typically several orders of magnitude lower. However, the aim of this study was to evaluate the performance of activated carbon adsorbents using artificially prepared PFAS solutions rather than to achieve ultra-trace detection limits required for regulatory monitoring. Several strategies could be employed to improve the sensitivity of the ^19^F-NMR method in future work, including increasing the number of scans, optimisation of pulse sequence parameters and relaxation delays, use of higher magnetic field strengths and cryogenically cooled probes, and refinement of the concentration of paramagnetic relaxation agents to enhance signal-to-noise ratios while minimising line broadening.

In addition, coupling quantitative ^19^F-NMR with a preconcentration step, such as solid-phase extraction using weak anion exchange resins, could substantially lower the effective detection limit and enable application to environmental samples, although large sample volumes would likely be required to achieve sufficiently high final PFAS concentrations.^42^ A key advantage of ^19^F-NMR in this context is the paucity of matrix interferences, which permits the preconcentration of large sample volumes without extensive clean-up or concern for co-extracted background constituents. Solid-phase extraction was not employed in the present study, as the method was intentionally designed to enable rapid screening of new adsorbents prior to more detailed analysis using conventional, higher-sensitivity techniques such as LC-MS/MS.

### 3.3. Adsorption performance

**Fig. 1:**
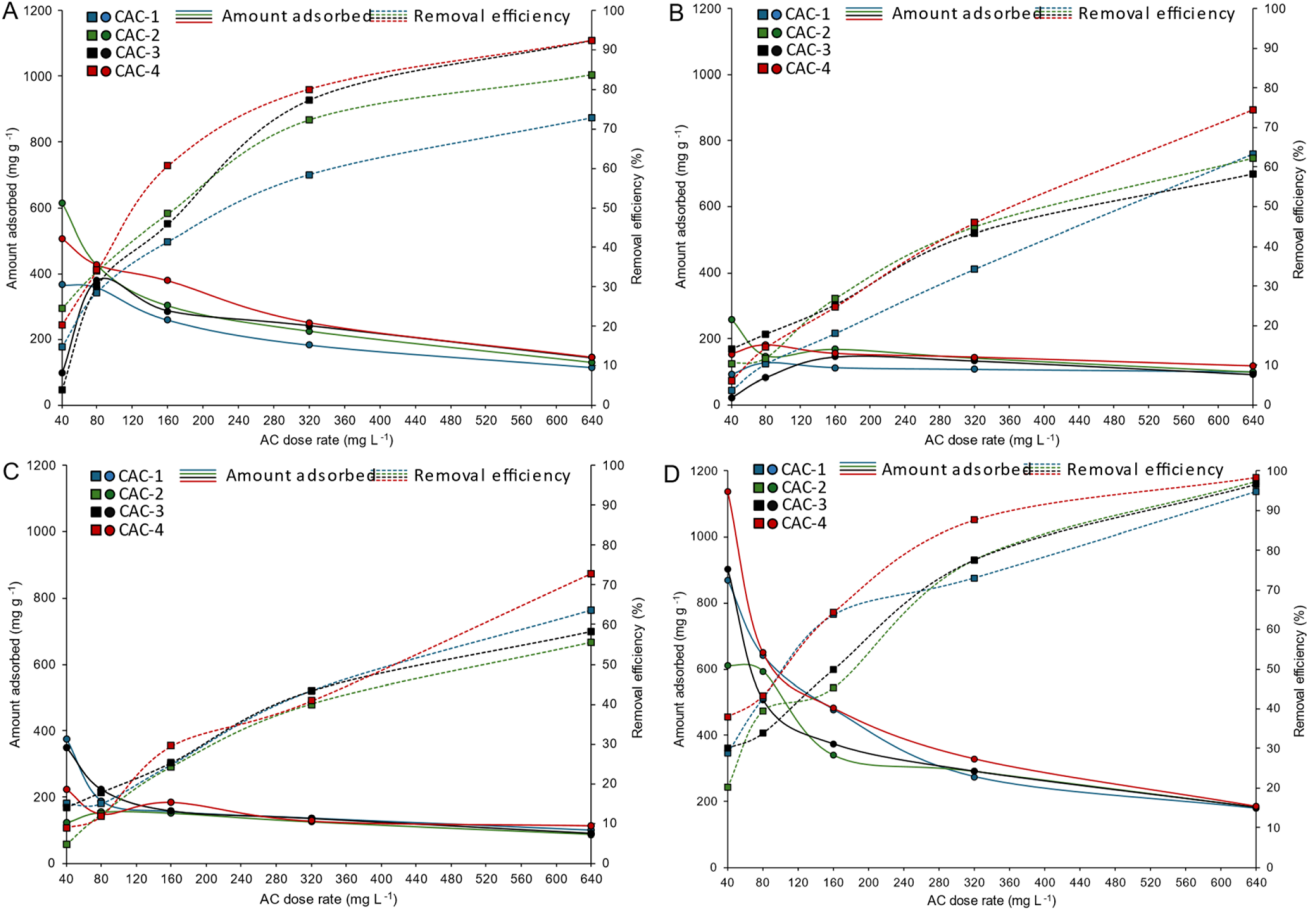
Adsorption capacities and removal efficiencies of (A) PFOA, (B) PFHxA, (C) PFBS and (D) PFOS, using the four ACs at varying dose rates.

**Fig. 2:**
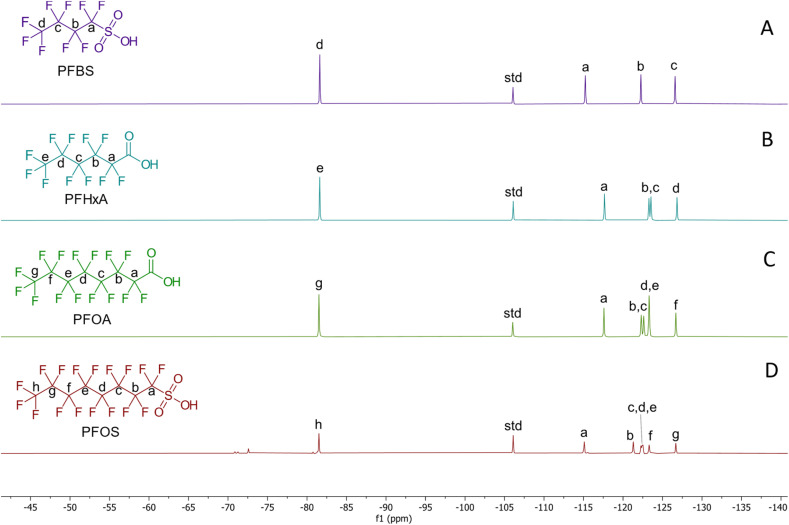
^19^F-NMR spectra of the (A) PFBS, (B) PFHxA, (C) PFOA and (D) PFOS. The standard peak (std) corresponds to the fluorine nuclei on 4,4′-difluorobenzophenone.

The adsorption capacities and removal efficiencies of the four activated carbons in ultrapure water are presented in Fig. 1, while representative ^19^F NMR spectra are shown in Fig. 2. Adsorption behaviour was quantified by normalising the integral of the PFAS –CF_3_ signal against that of the internal standard (4,4′-difluorobenzophenone). Freundlich, Langmuir and Sips models were applied to the adsorption data, and the corresponding fitting parameters are reported in Table S1, with model fits shown in Fig. S1–S4.

Adsorption experiments were conducted by varying the adsorbent dose at a fixed PFAS concentration, rather than by varying solute concentration at a fixed adsorbent mass. Therefore, the isotherm parameters should be viewed as practical indicators of how adsorption changes with dose, rather than strict thermodynamic constants. Under these conditions, direct comparison of apparent *q*_max_ values across different PFAS species is inherently limited, and adsorption performance is therefore primarily assessed based on experimentally observed adsorption capacities and removal efficiencies (Fig. 1).

Across all activated carbons, adsorption performance increased with increasing PFAS carbon chain length, with PFOS and PFOA exhibiting substantially higher adsorption capacities and removal efficiencies than PFHxA and PFBS under equivalent dose conditions (Fig. 1). This trend is consistent with the normalised ^19^F NMR signal intensities shown in Fig. 2 and reflects the increasing contribution of hydrophobic interactions between the fluorinated carbon chain and the graphitic surfaces of activated carbon as PFAS chain length increases.

Short-chain PFAS, particularly PFBS, displayed unfavourable adsorption behaviour across all activated carbons. As shown in Fig. 1C and the corresponding isotherm fits (Fig. S3), PFBS exhibited low adsorption capacities and weak sensitivity to increasing adsorbent dose, resulting in poorly constrained model parameters and reduced goodness-of-fit values in Table S1. This behaviour reflects the higher aqueous solubility and reduced hydrophobic character of short-chain PFAS, which limits their interaction with activated carbon surfaces and highlights a known limitation of activated carbon treatment technologies.

Among the activated carbons studied, CAC-4 consistently exhibited the highest adsorption performance for all PFAS investigated, followed by CAC-3, CAC-2 and CAC-1 (Fig. 1). This ranking correlates strongly with the measured textural and chemical properties of the adsorbents (Tables 4 and 5). CAC-4 possessed the highest mesoporosity and the greatest concentration of surface oxygen-containing functional groups, providing a larger number of accessible adsorption sites. While electrostatic attraction between negatively charged PFAS head groups and positively charged surface sites may contribute to adsorption under the experimental pH conditions, the preferential adsorption of long-chain PFAS indicates that hydrophobic interactions are the dominant adsorption mechanism.

**Fig. 3:**
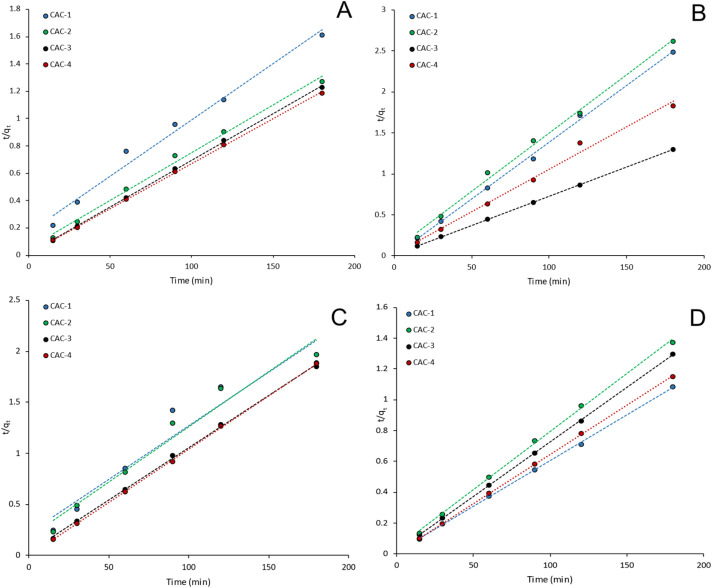
The pseudo-second order adsorption kinetics model provides the best fit for the adsorption of (A) PFOA, (B) PFHxA, (C) PFBS and (D) PFOS, with all adsorbents.

Adsorption kinetics further support the trends observed in the equilibrium experiments. Kinetic data for all PFAS–adsorbent systems were best described by the pseudo-second-order model (Fig. 3), indicating that adsorption rate behaviour is governed predominantly by surface interactions rather than simple mass transfer processes. The kinetic profiles are presented up to 200 minutes to emphasise early-stage adsorption behaviour and enable comparison of adsorption rates between adsorbents, while extended contact times (48 h) were used to confirm equilibrium attainment. Similar adsorption rates observed for certain activated carbons are consistent with their comparable textural properties and surface chemistries.

## 4 Conclusions

In the present study, the simple use of a new ^19^F-NMR method has been demonstrated and is applicable to the screening of new activated carbon adsorbents for the removal of PFAS from water. The ^19^F-NMR adsorption data combined with the extensive physical and chemical characterisation data for the four ACs enabled the understanding of the adsorption mechanisms taking place between the PFAS and the ACs. Consequently, AC's tendency to preferentially adsorb long chain PFAS was confirmed by this study, with results comparable to similar studies where more traditional analytical methods, like LC-MS/MS, were employed. Lastly, the evaluation of total PFAS removal is viable with the presented ^19^F-NMR method, as shown by the adsorption experiments using the mixtures of PFOA and PFBS (Fig. S9).

CAC-4 proved to be highest-performing adsorbent for the removal of all the PFAS tested in this study, owing to its high surface area, greater O : C ratio and high mesoporosity. Exhibiting high mesoporosity meant there was greater access for the PFAS to bind to the adsorption sites. The greater concentration of surface oxygen-containing functional groups may have contributed to better adsorption of the short-chain PFAS *via* the formation of a greater number of hydrogen bonds between the negatively charged polar head groups of the PFAS and the protonated oxygen functional groups of the AC, a mechanism described by Lenka *et al.*^43^

The advantages of ^19^F-NMR over LC-MS for the rapid evaluation of new adsorbents have been demonstrated, with the experiments requiring no sample clean-up, fast experiment time, and only requiring a single inexpensive internal standard. Preliminary adsorption experiments could be conducted with ^19^F-NMR to short-list adsorbents that can then be further analysed with techniques like LC-MS, saving valuable time.

## Conflicts of interest

There are no conflicts of interest to declare.

## Supplementary Material

RA-016-D5RA06437F-s001

## Data Availability

All data supporting the findings of this study are available within the article and its supplementary information (SI). Supplementary information is available. See DOI: https://doi.org/10.1039/d5ra06437f.
