# Supplementary material for: Analysis of per- and polyfluoroalkyl substances (PFAS) removal with activated carbon using 19F nuclear magnetic resonance spectroscopy
Source: RSC Adv. 2026 Jan 26;16(7):5716–25. doi: 10.1039/d5ra06437f (PMC12833916; doi:10.1039/d5ra06437f)
Supplement: RA-016-D5RA06437F-s001 [file RA-016-D5RA06437F-s001.pdf]

Supplementary Information (SI) for RSC Advances.
This journal is © The Royal Society of Chemistry 2026

Supplementary Information

Adsorbents	Freundlich			Langmuir			Sips			
	K_F	n_F	R^2	K_L	q_{max}	R^2	K_s	q_{max}	n_S	R^2
	PFOA									
CAC-1	3.34	0.93	0.996	8×10^{-4}	5560	0.951	0.0173	543.3	2.09	0.977
CAC-2	4.07	0.88	0.989	5×10^{-4}	14400	0.987	6.45×10^{-7}	4.40×10^{7}	1.14	0.909
CAC-3	37.96	1.84	0.994	0.023	622	0.992	1.81×10^{-8}	6.29×10^{5}	0.54	0.925
CAC-4	40.02	1.66	0.992	0.018	923	0.991	2.69×10^{-8}	1.48×10^{6}	0.60	0.927
	PFBS									
CAC-1	0.02	0.41	0.997	2×10^{-4}	29300	0.938	1.27×10^{-4}	5.32×10^{7}	2.46	0.943
CAC-2	0.001	0.31	0.990	2×10^{-4}	35000	0.903	5.12×10^{-4}	1.25×10^{8}	3.67	0.136
CAC-3	4×10^{-4}	0.29	0.952	3×10^{-4}	30200	0.864	3.77×10^{-3}	1.04×10^{8}	8.30	0.638
CAC-4	0.003	0.34	0.957	2×10^{-4}	34400	0.908	4.50×10^{-4}	4.37×10^{7}	3.19	0.776
	PFHxA									
CAC-1	2×10^{-4}	0.29	0.969	3×10^{-6}	1.83×10^{6}	0.900	0.00161	8.95×10^{7}	5.49	0.867
CAC-2	0.32	0.32	0.944	5×10^{-6}	1.41×10^{6}	0.892	0.00043	4.11×10^{7}	3.12	0.950
CAC-3	0.41	0.41	0.977	3×10^{-5}	2.08×10^{5}	0.938	0.00013	5.32×10^{7}	2.46	0.832
CAC-4	0.34	0.34	0.976	4×10^{-5}	1.93×10^{3}	0.933	0.00048	9.71×10^{7}	3.50	0.796
	PFOS									
CAC-1	66.86	2.50	0.990	0.05	475	0.988	5.41×10^{-4}	2.02×10^{3}	0.46	0.884
CAC-2	157.03	12.34	0.923	0.62	230	0.928	0.328	224.2	6.97	0.751
CAC-3	90.18	3.52	0.978	0.15	324	0.972	7.60×10^{-9}	1.94×10^{4}	0.29	0.867
CAC-4	87.20	2.43	0.976	0.05	649	0.965	8.04×10^{-9}	1.87×10^{5}	0.41	0.875

Table S1. Freundlich, Langmuir and Sips parameters associated with CAC-1, CAC-2, CAC-3 and CAC-4. Where K_F is the Freundlich constant [(mg g^{-1})(L mg^{-1})$^{1/n}$], n_F is the dimensionless heterogeneity factor, K_L is the Langmuir constant (L mg^{-1}), q_{max} is the maximum adsorption capacity for the adsorbent (mg g^{-1}), K_S is the Sips constant (L mg^{-1}), n_S is the dimensionless Sips heterogeneity factor and R^2 is the coefficient of determination.

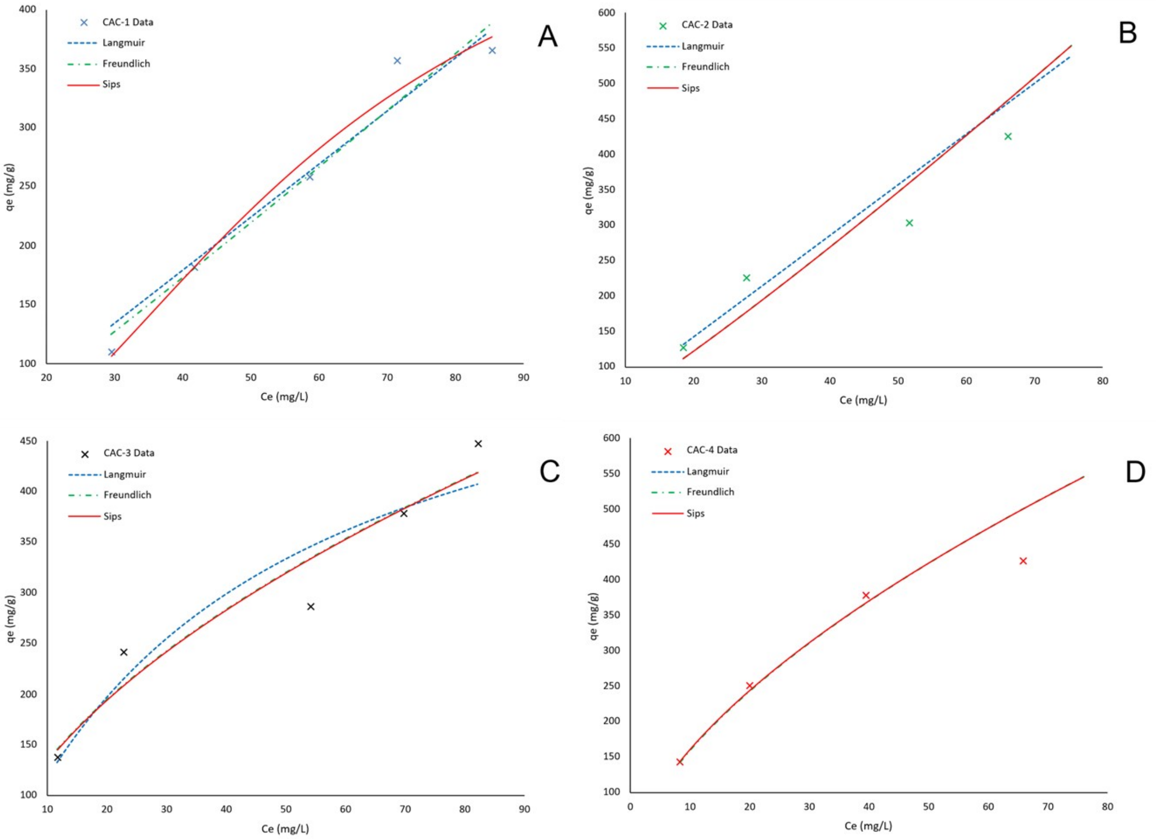

Figure S1. Langmuir, Freundlich and Sips adsorption isotherm models for the adsorption of PFOA on (A) CAC-1, (B) CAC-2, (C) CAC-3, and (D) CAC-4.

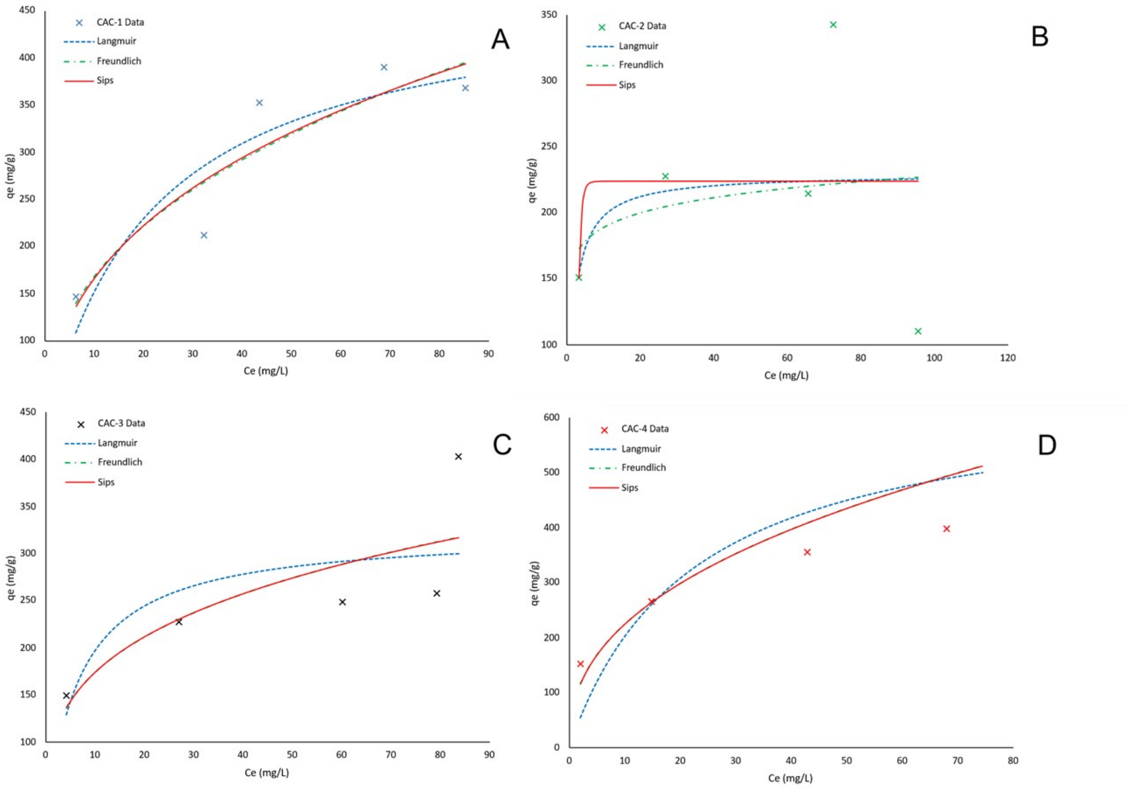

Figure S2. Langmuir, Freundlich and Sips adsorption isotherm models for the adsorption of PFOS on (A) CAC-1, (B) CAC-2, (C) CAC-3, and (D) CAC-4.

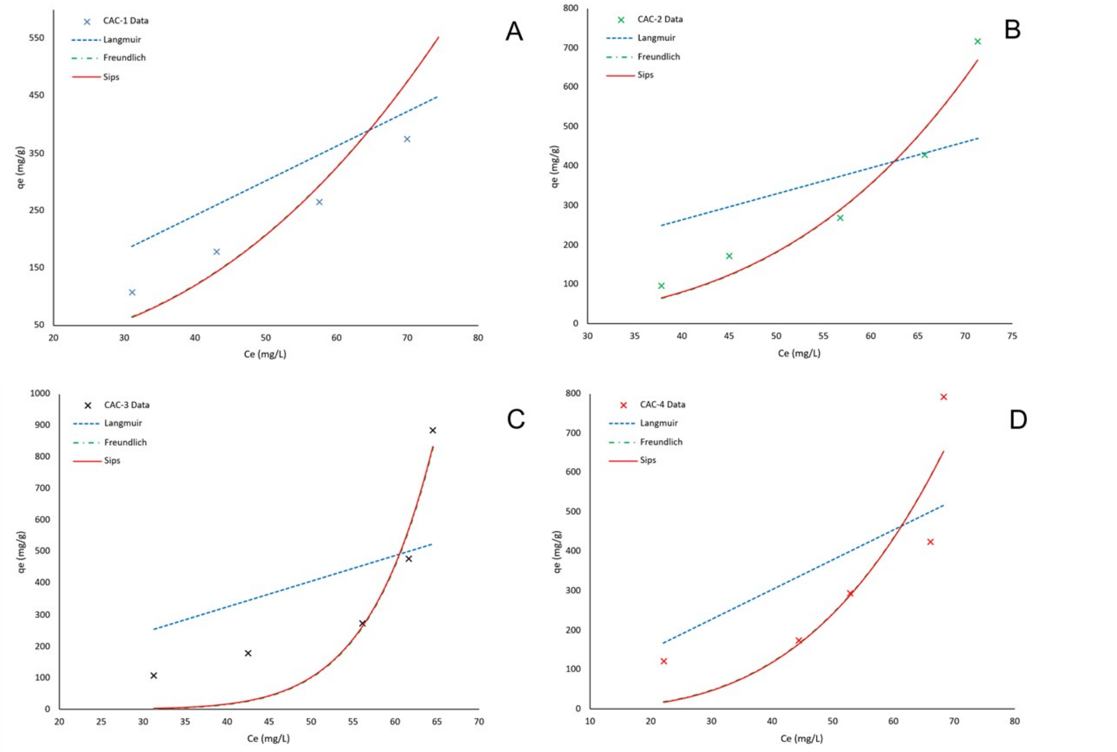

Figure S3. Langmuir, Freundlich and Sips adsorption isotherm models for the adsorption of PFBS on (A) CAC-1, (B) CAC-2, (C) CAC-3, and (D) CAC-4.

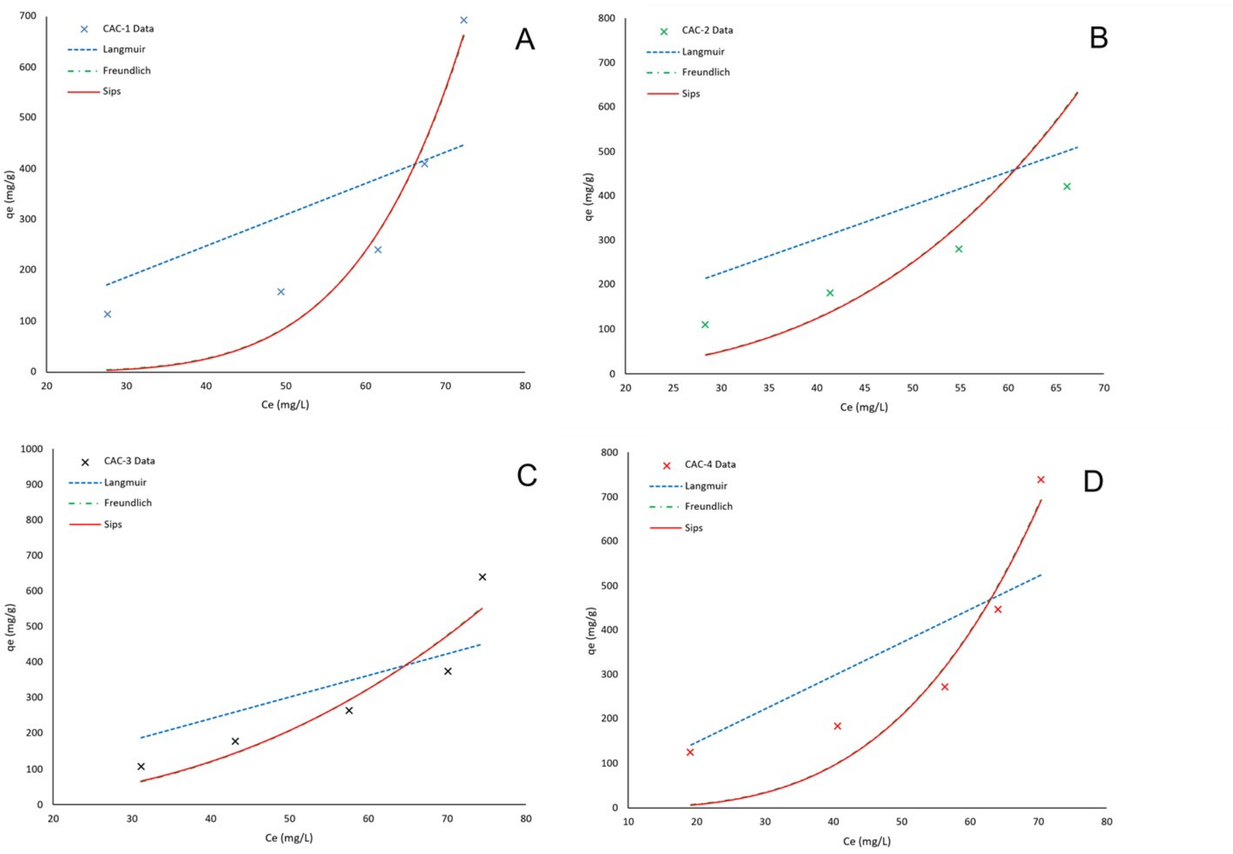

Figure S4. Langmuir, Freundlich and Sips adsorption isotherm models for the adsorption of PFHxA on (A) CAC-1, (B) CAC-2, (C) CAC-3, and (D) CAC-4.

Figure S5. Deconvoluted C1s XPS spectra of CAC-1, CAC-2, CAC-3 and CAC-4

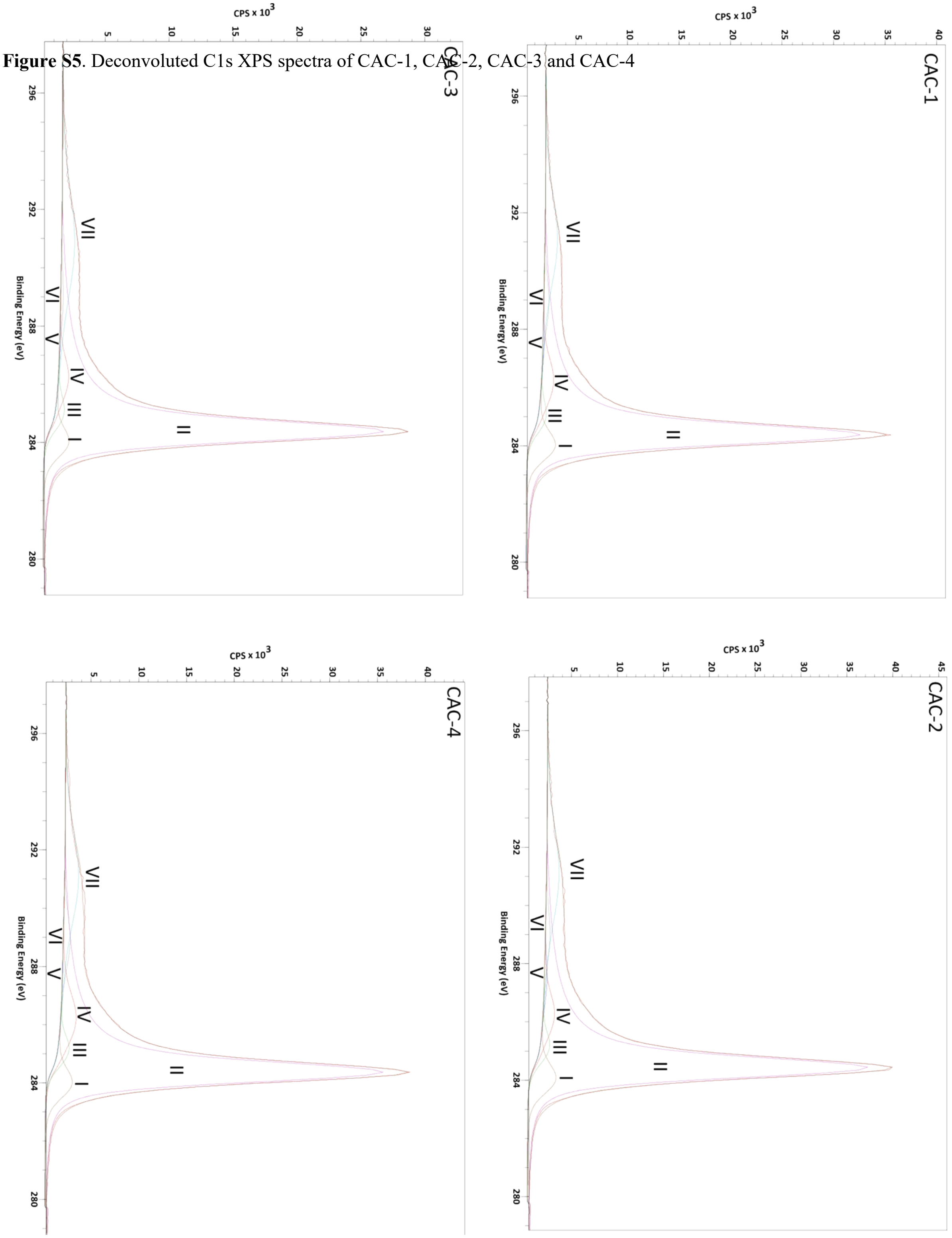

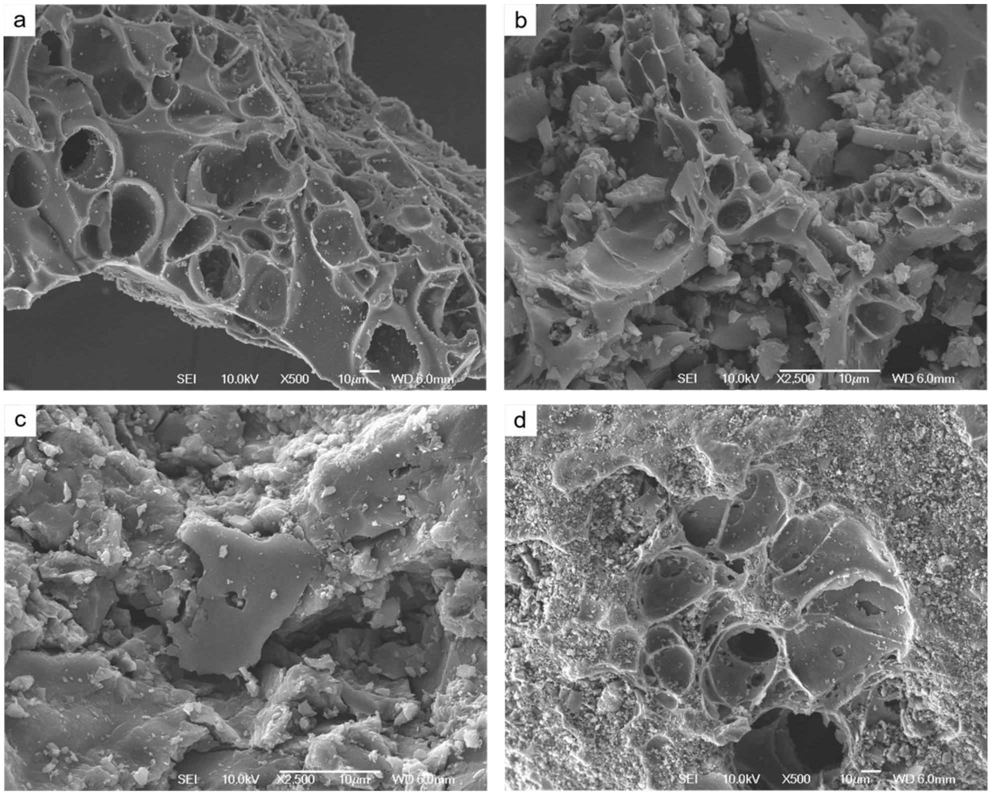

Figure S6. SEM images of the four activated carbons; (a) CAC-1, (b) CAC-2, (c) CAC-3 and (d) CAC-4.

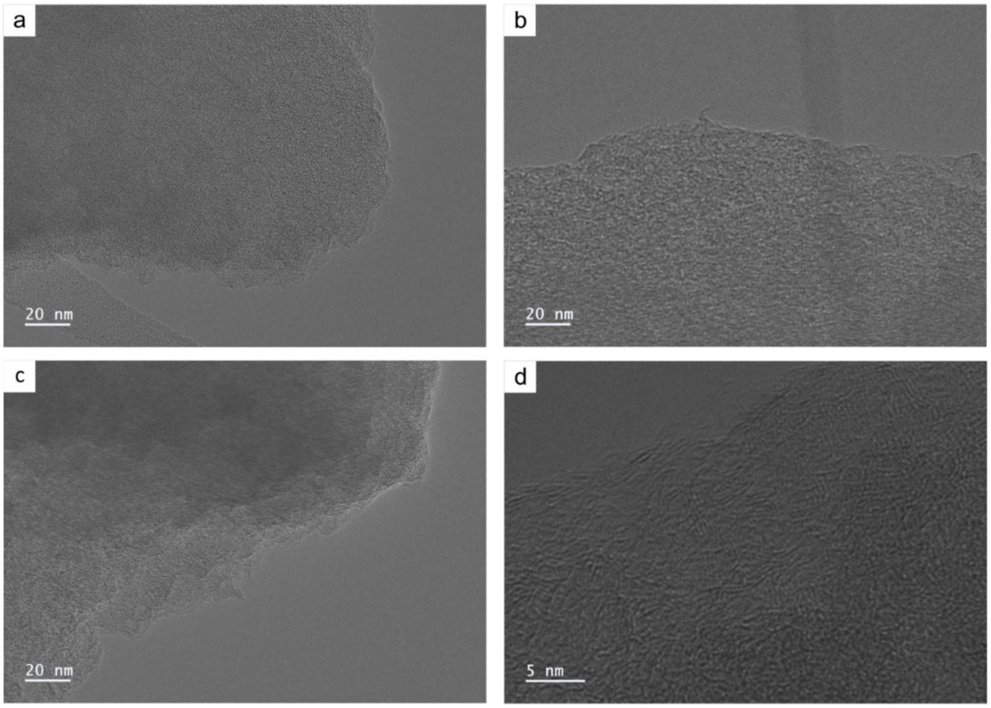

Figure S7. TEM images of the four activated carbons; (a) CAC-1, (b) CAC-2, (c) CAC-3 and (d) CAC-4.

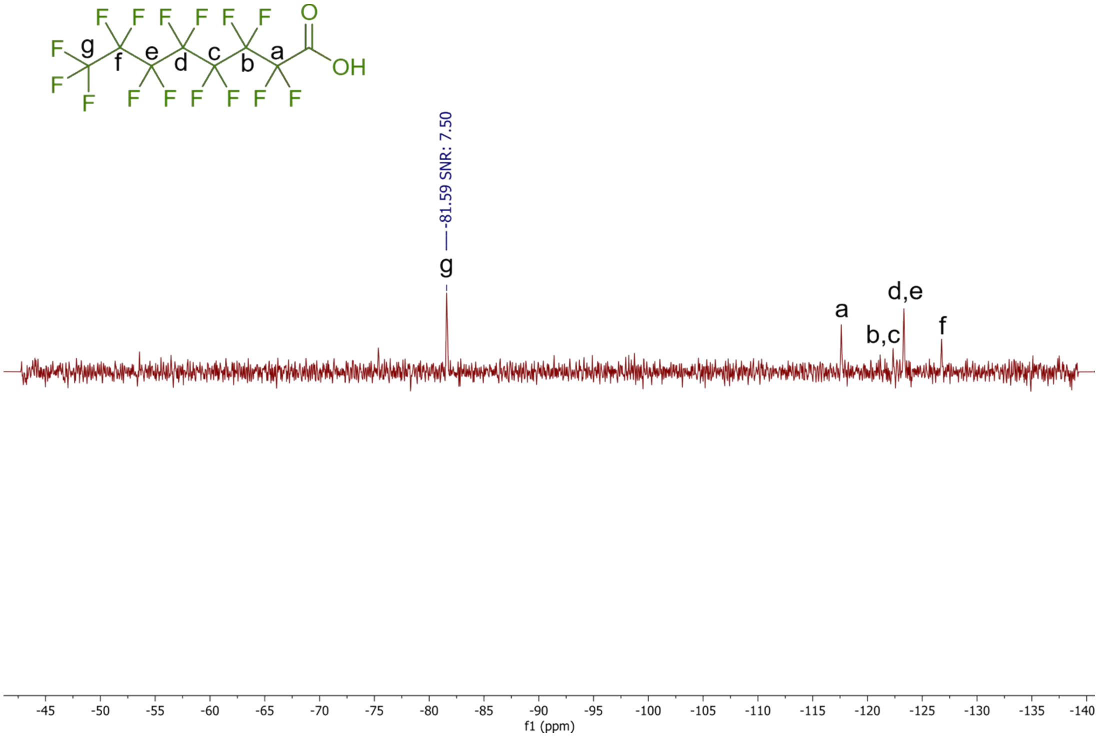

Figure S8. ^{19}F NMR spectrum of PFOA at 1 mg L^{-1} in 1:1 ratio $H_2O:CD_3OD$ showing the -CF3 peak at -81.59 ppm (g) with a signal to noise ratio of > 3.

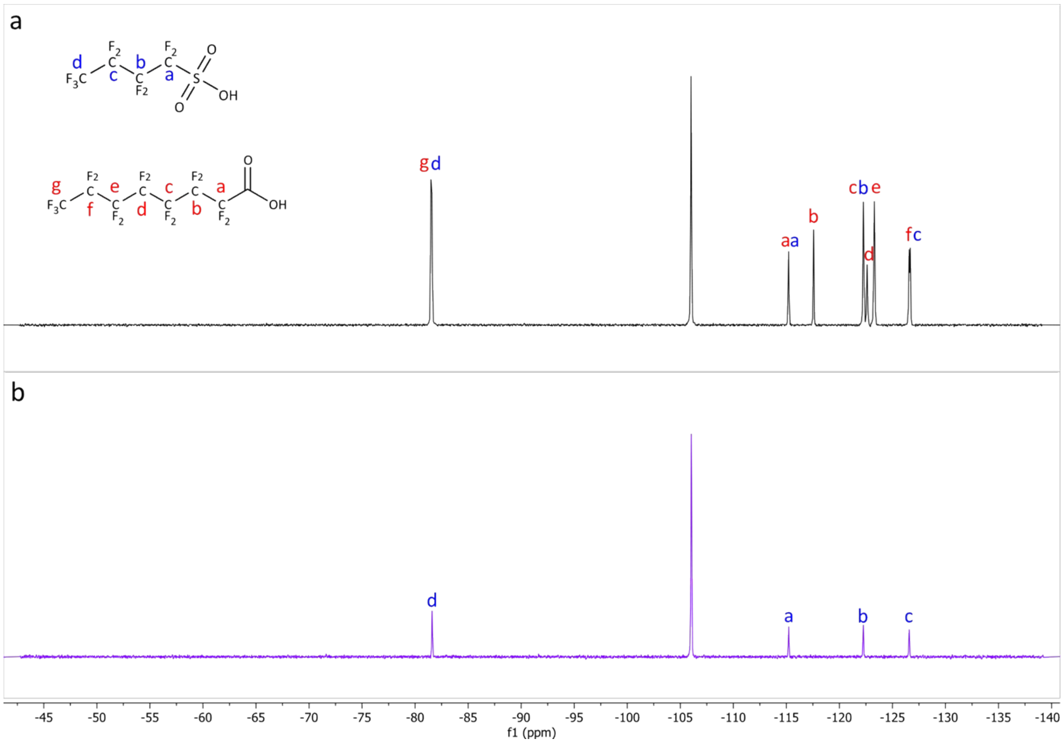

Figure S9. ^{19}F NMR spectra of a mixed solution of PFBS and PFOA (a) before treatment with CAC-4, and (b) after treatment with CAC-4, showing how total PFAS can be quantified using the $-CF_3$ signal.

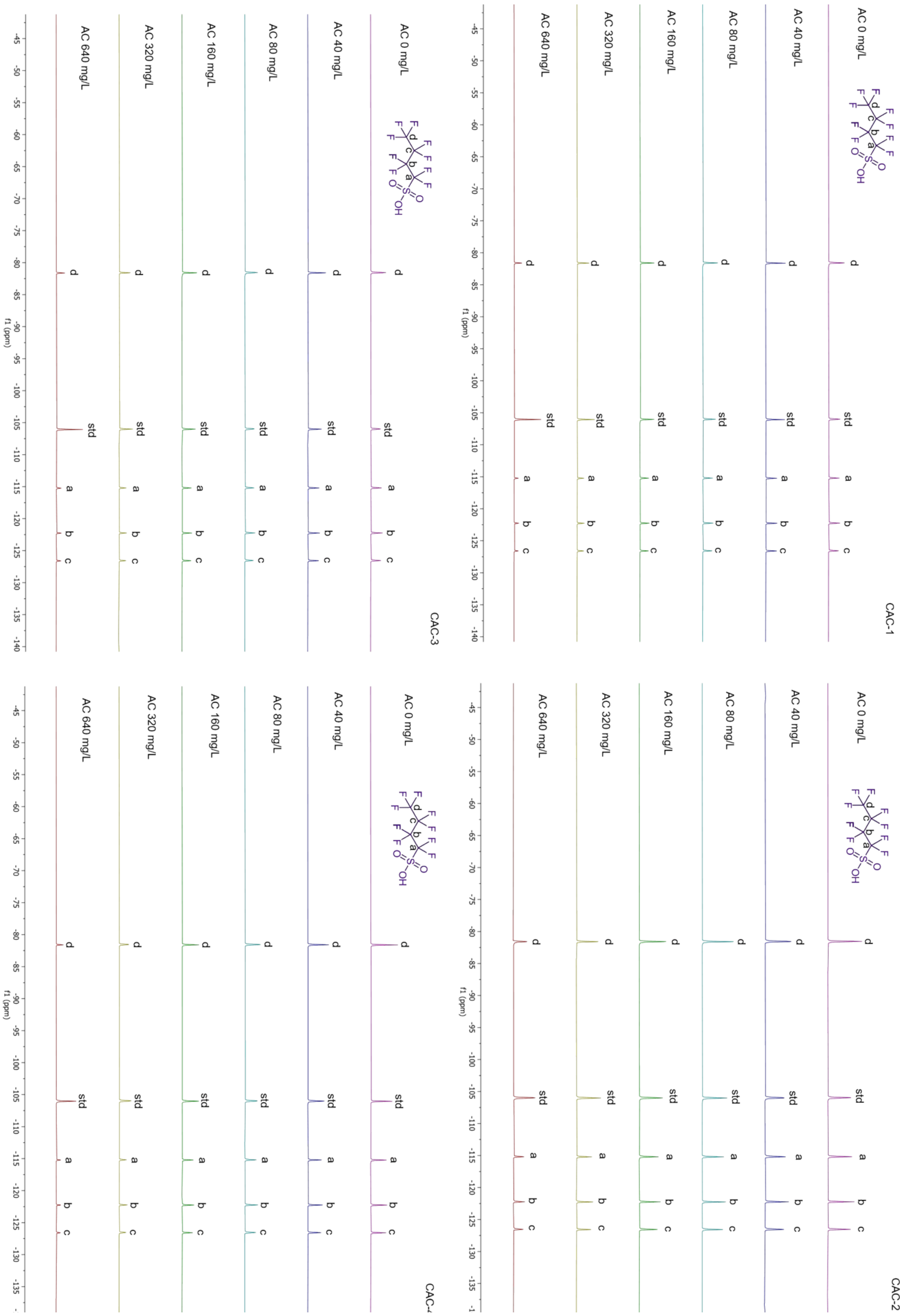

Figure S10. Stacked NMR spectra obtained for the adsorption isotherms of PFBS with CAC-1, CAC-2, CAC-3 and CAC-4.

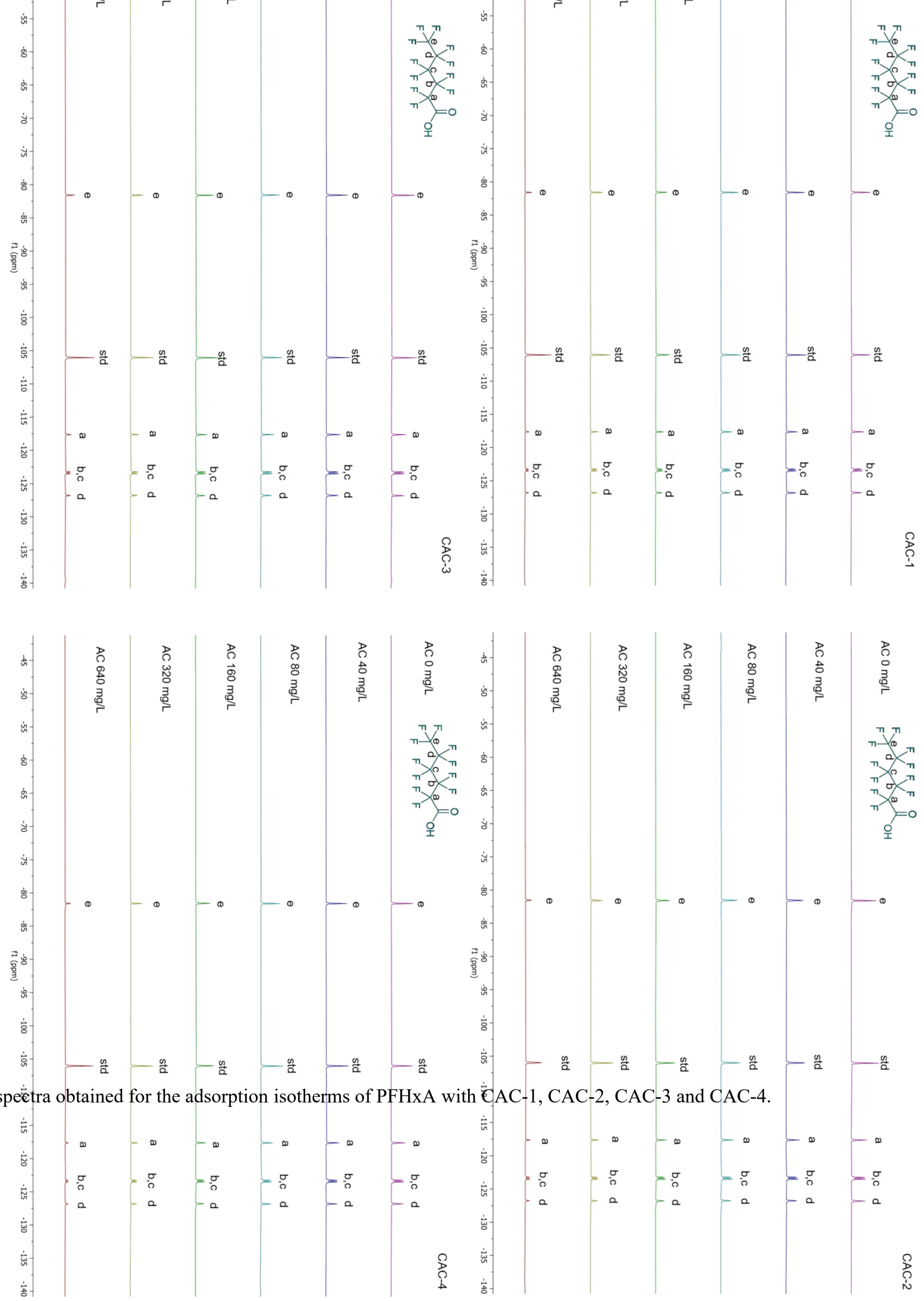

Figure S11. Stacked NMR spectra obtained for the adsorption isotherms of PFHxA with CAC-1, CAC-2, CAC-3 and CAC-4.

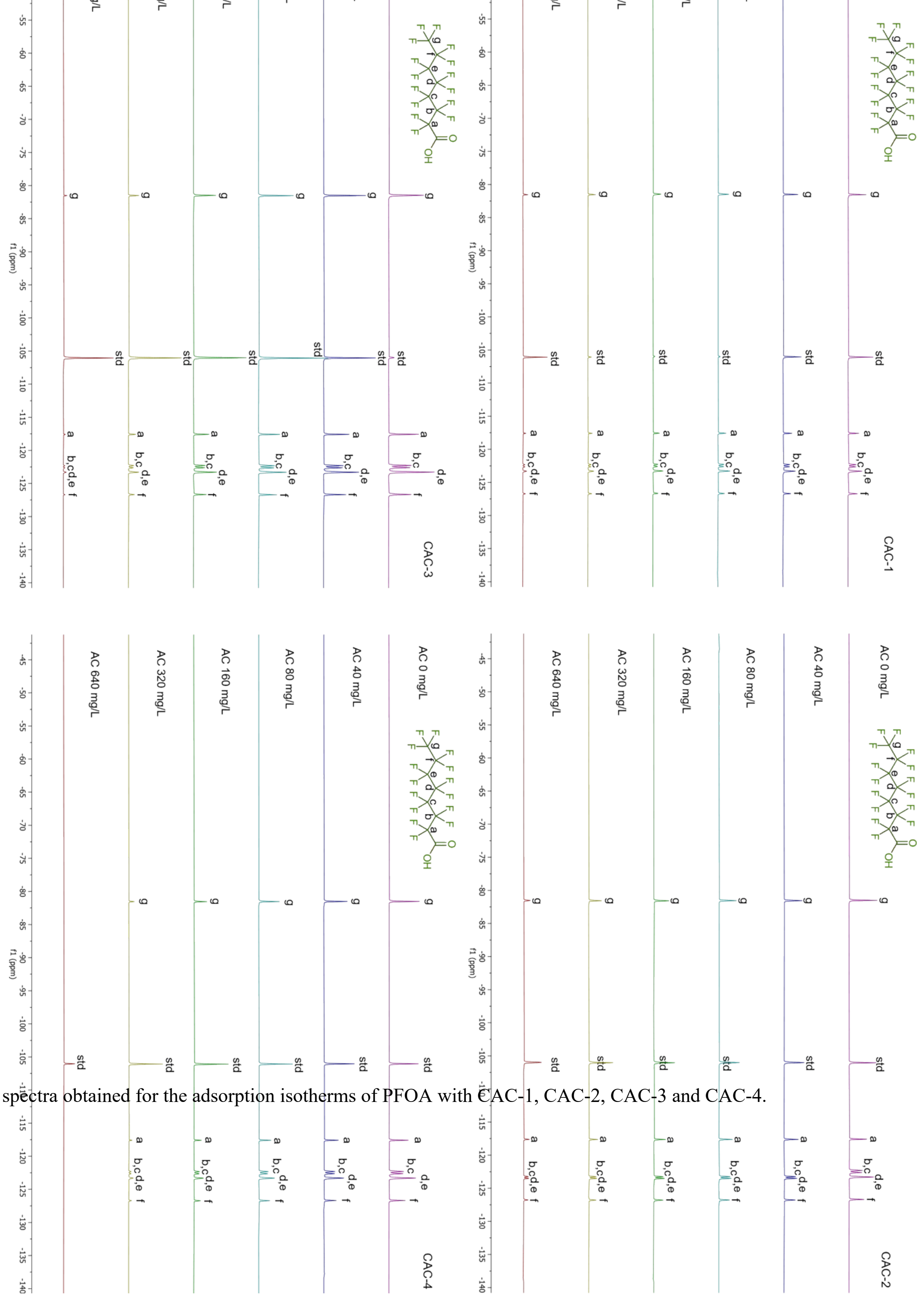

Figure S12. Stacked NMR spectra obtained for the adsorption isotherms of PFOA with CAC-1, CAC-2, CAC-3 and CAC-4.

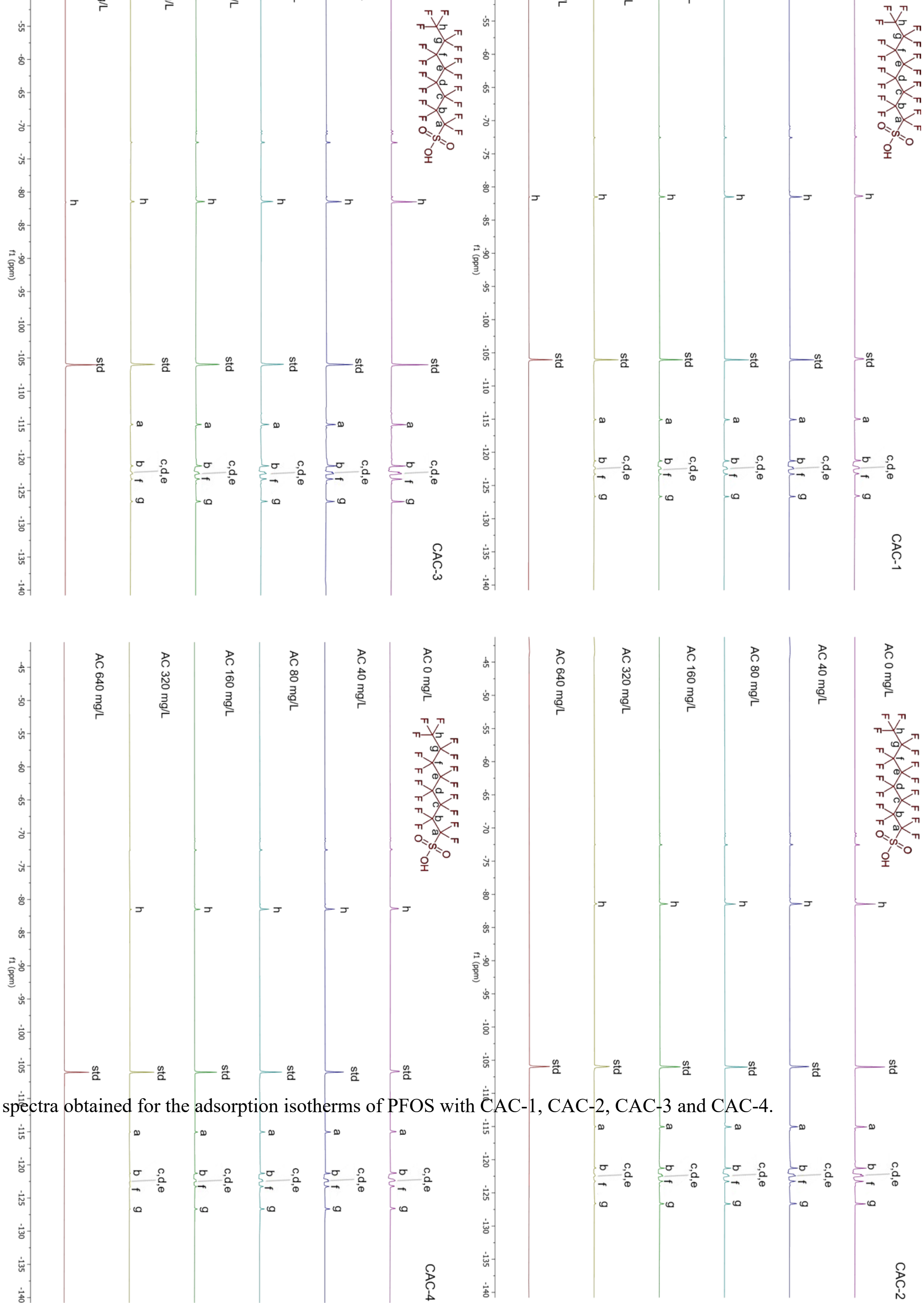

Figure S13. Stacked NMR spectra obtained for the adsorption isotherms of PFOS with CAC-1, CAC-2, CAC-3 and CAC-4.

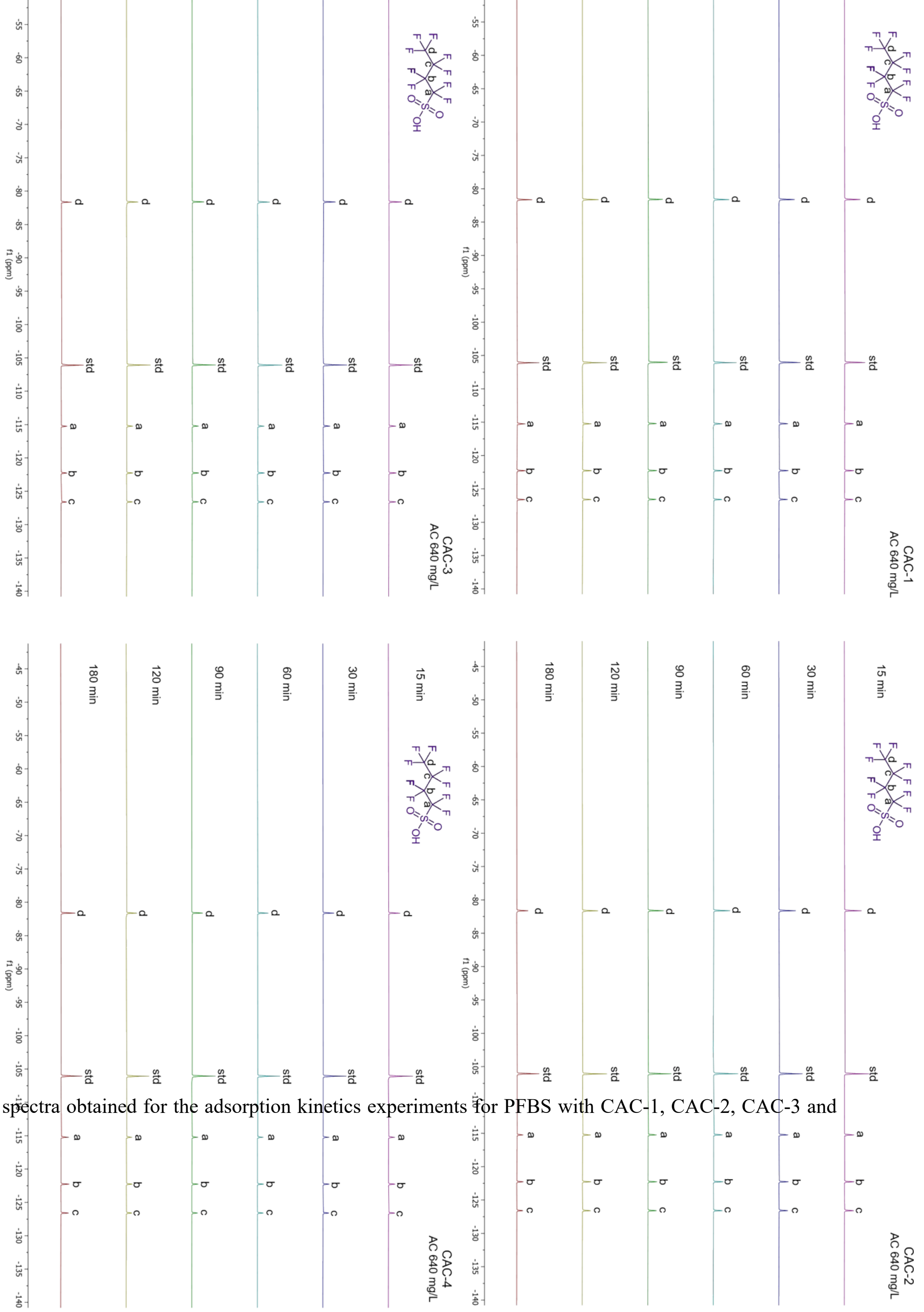

Figure S14. Stacked NMR spectra obtained for the adsorption kinetics experiments for PFBS with CAC-1, CAC-2, CAC-3 and CAC-4.

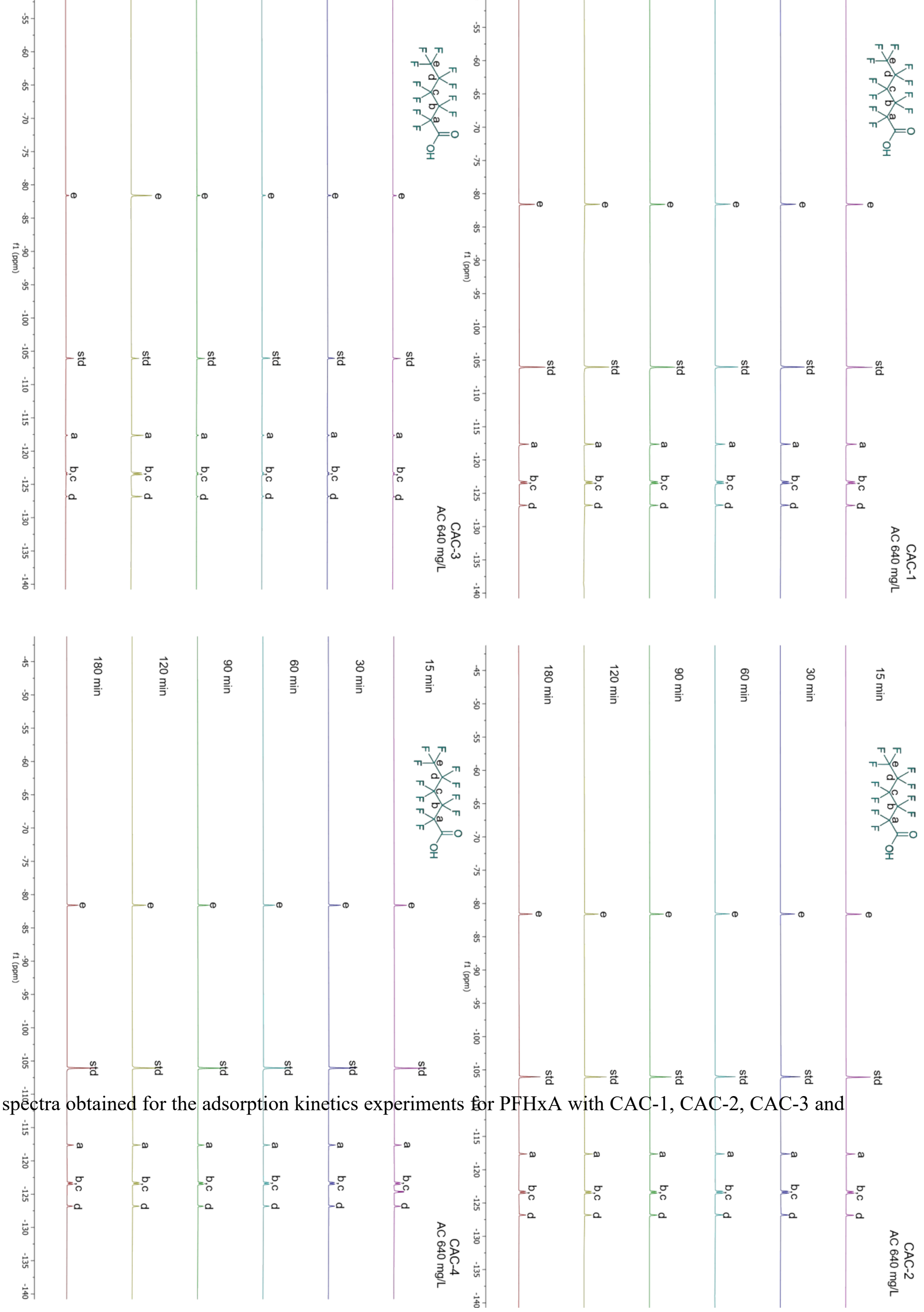

Figure S15. Stacked NMR spectra obtained for the adsorption kinetics experiments for PFHxA with CAC-1, CAC-2, CAC-3 and CAC-4.

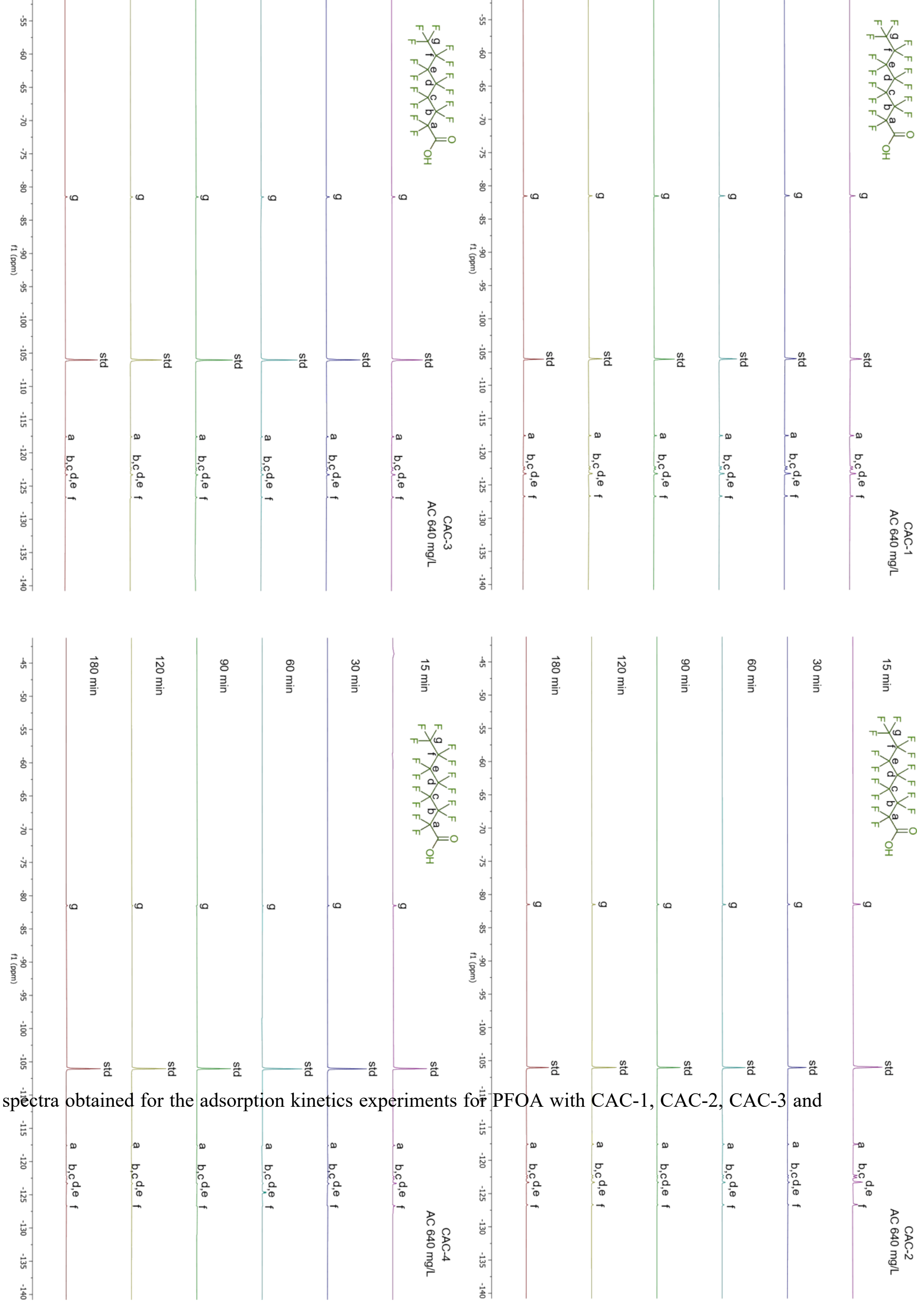

Figure S16. Stacked NMR spectra obtained for the adsorption kinetics experiments for PFOA with CAC-1, CAC-2, CAC-3 and CAC-4.

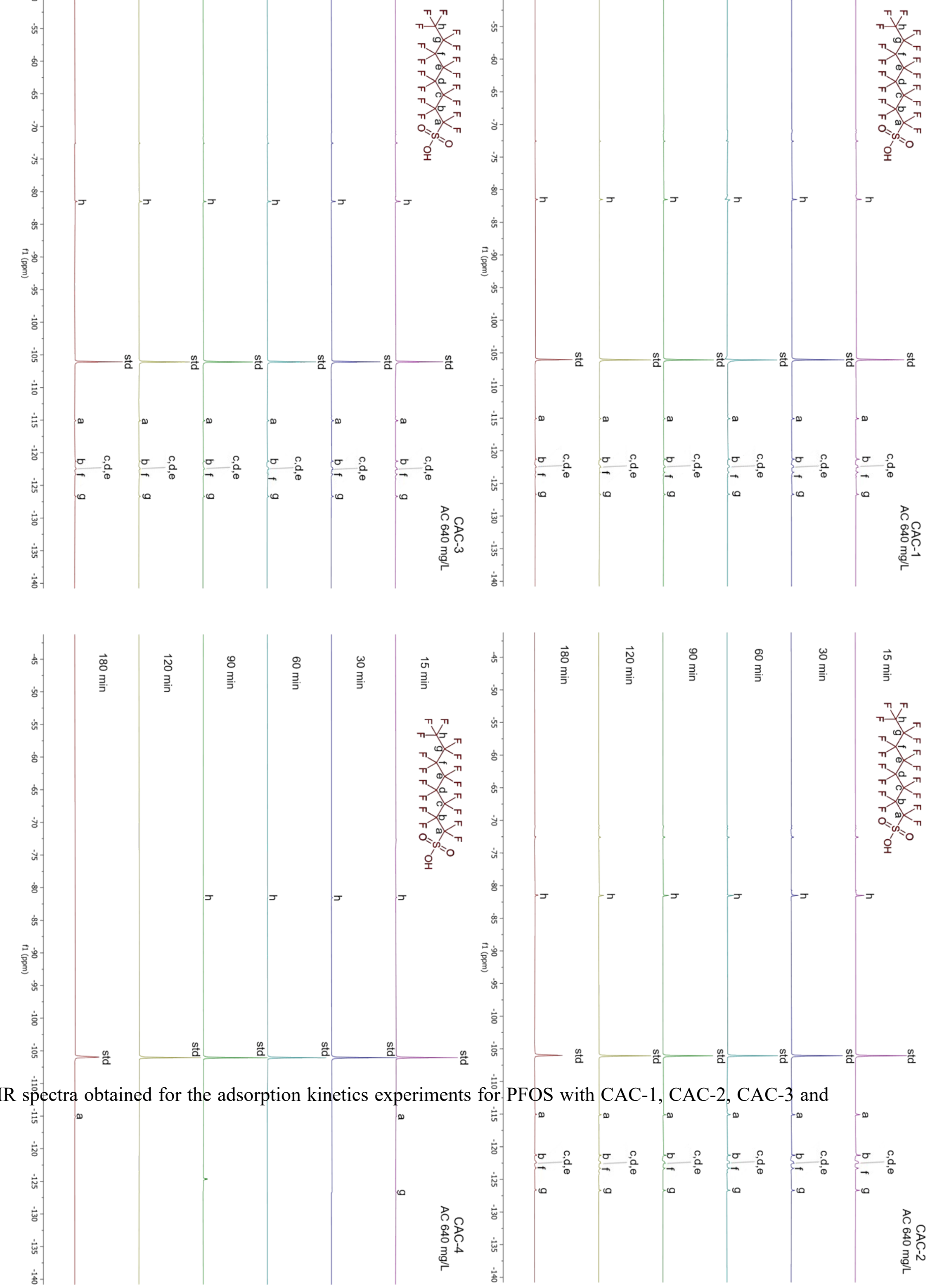

Figure S17. Stacked NMR spectra obtained for the adsorption kinetics experiments for PFOS with CAC-1, CAC-2, CAC-3 and CAC-4.
